# Microbioreactor Array Screening of Wnt Modulators and Microenvironmental Factors in Osteogenic Differentiation of Mesenchymal Progenitor Cells

**DOI:** 10.1371/journal.pone.0082931

**Published:** 2013-12-23

**Authors:** Jessica E. Frith, Drew M. Titmarsh, Harish Padmanabhan, Justin J. Cooper-White

**Affiliations:** 1 Australian Institute for Bioengineering & Nanotechnology, The University of Queensland, St. Lucia, Queensland, Australia; 2 School of Chemical Engineering, The University of Queensland, St. Lucia, Queensland, Australia; 3 Materials Science and Engineering Division, CSIRO, Clayton, Victoria, Australia; University of Udine, Italy

## Abstract

Cellular microenvironmental conditions coordinate to regulate stem cell populations and their differentiation. Mesenchymal precursor cells (MPCs), which have significant potential for a wide range of therapeutic applications, can be expanded or differentiated into osteo- chondro- and adipogenic lineages. The ability to establish, screen, and control aspects of the microenvironment is paramount if we are to elucidate the complex interplay of signaling events that direct cell fate. Whilst modulation of Wnt signaling may be useful to direct osteogenesis in MPCs, there is still significant controversy over how the Wnt signaling pathway influences osteogenesis. In this study, we utilised a full-factorial microbioreactor array (MBA) to rapidly, combinatorially screen several Wnt modulatory compounds (CHIR99021, IWP-4 and IWR-1) and characterise their effects upon osteogenesis. The MBA screening system showed excellent consistency between donors and experimental runs. CHIR99021 (a Wnt agonist) had a profoundly inhibitory effect upon osteogenesis, contrary to expectations, whilst the effects of the IWP-4 and IWR-1 (Wnt antagonists) were confirmed to be inhibitory to osteogenesis, but to a lesser extent than observed for CHIR99021. Importantly, we demonstrated that these results were translatable to standard culture conditions. Using RT-qPCR of osteogenic and Wnt pathway markers, we showed that CHIR exerted its effects via inhibition of *ALP* and *SPP1* expression, even though other osteogenic markers (*RUNX2*, *MSX2*, *DLX, COL1A1)* were upregulated. Lastly, this MBA platform, due to the continuous provision of medium from the first to the last of ten serially connected culture chambers, permitted new insight into the impacts of paracrine signaling on osteogenic differentiation in MPCs, with factors secreted by the MPCs in upstream chambers enhancing the differentiation of cells in downstream chambers. Insights provided by this cell-based assay system will be key to better understanding signaling mechanisms, as well as optimizing MPC growth and differentiation conditions for therapeutic applications.

## Introduction

Mesenchymal stem cells (MSCs) are attractive candidates for a wide range of tissue engineering and regenerative medicine applications due to their availability and multi-lineage differentiation potential (including osteogenic, chondrogenic and adipogenic lineages), as well as their immunosuppressive properties [1], [2], [3]. It is therefore desirable to develop a good understanding of the signaling mechanisms that guide their behavior so that cellular activity can be appropriately directed towards specific outcomes for therapeutic purposes.

It is widely recognised that key developmental signaling pathways, including those involving bone morphogenetic protein (BMP), fibroblast growth factor (FGF), and wingless (Wnt), have a critical role to play in MSC biology, with a complex interplay of signaling through these pathways coordinating both proliferation and lineage specification [4]. However, although much has been elucidated about the roles of different signaling mechanisms in MSC fate, many conclusions have been confounded by the fact that the cellular response is critically dependent upon microenvironmental parameters, such as cell density at the onset of differentiation, the timing of exposure to inductive signals, and the impacts of autocrine/paracrine signaling [5], [6], [7]. These factors, amongst others, have resulted in conflicting reports regarding the activities of many signaling pathways.

Given the significant parameter space of factors known to affect the cellular microenvironment, in order to truly gain greater understanding of the significance of these signaling mechanisms and how their activity may be influenced by changes in such microenvironmental conditions, we require systems or tools that allow for a more high-throughput, combinatorial approach. We have previously developed a microbioreactor array (MBA) platform which delivers a full factorial set of factors – three concentrations each of three different factors – to cells under continuous flow [8], [9]. This continuous perfusion microbioreactor also allows progressive accumulation of paracrine factors through serially-connected culture chambers, permitting spatially-segregated assessment of their impact. Such a system has significant advantages over conventional culture techniques, in that it readily provides combinatorial media formulations (for example combining activators or inhibitors of target signaling pathways), generating data for numerous conditions in parallel whilst utilizing reduced cell numbers and amounts of reagents. By leveraging technologies such as this it is possible to examine large parameter spaces to determine how different signaling pathways may cooperatively influence MSC growth and differentiation under various microenvironmental conditions. This information can then be related to the conditions relevant to particular therapeutic applications.

Wnt signaling, which has been shown to play an important role in directing MSC behavior, is one such mechanism that highlights the complexity of elucidating the effects of signaling upon MSC fate. This particular mechanism has attracted significant interest in recent times, both in terms of the development of pharmaceutical targets, as well as in the development of protocols to direct MSC differentiation for regenerative medicine. The Wnts are a family of evolutionarily conserved glycoproteins, with 19 family members in humans. Wnt signals are received upon Wnt binding to the cell surface co-receptors Frizzled (Fzd) and low-density-lipoprotein receptor-related protein (LRP)-5 and 6. The resulting signal can be transduced by a number of mechanisms; canonical Wnt signaling in which stabilization of β-catenin causes it to accumulate and translocate to the nucleus of the cell where it activates transcription of target genes, or non-canonical mechanisms not involving β-catenin but instead acting through jun N-terminal kinase (JNK) or calcium signaling.

Human MSCs (hMSCs) have shown that they express all the necessary molecular machinery for Wnt signaling [10], but there are only a small number of publications that have probed the effect of canonical and non-canonical Wnt signaling on the proliferation and differentiation potential of MSC’s. For example, canonical Wnt signaling was shown to play an important role in maintaining MSCs in an undifferentiated and proliferative state [11], [12], [13]. On the contrary, there are also reports which show that canonical Wnt signaling promotes the differentiation of MSCs [14], [15], [16]. Other reports have shown that non-canonical Wnt has no effect on proliferation but enhances differentiation potential of MSCs in a reversible manner (i.e. upon removal of non-canonical Wnt proteins) [17]. These conflicting reports on the relative impacts of canonical and non-canonical Wnt signaling are to be contextualized with the statement that each of these studies have utilised different agonist or antagonist molecules (such as Wnt 3a, a canonical Wnt Agonist or Wnt 5a, a non-canonical Wnt agonist), at differing concentrations and varied temporal provision, and with different MSC sources (or species), along with them covering a range of both *in vitro* and *in vivo* models [11], [18].

This situation provided us with the necessary motivation to utilise the MBA system as a tool to test a wide range of combinations of a panel of three well characterized small molecule Wnt activators and inhibitors in MSCs undergoing osteogenesis, and thereafter relate the osteogenic outcomes back to the underlying signals. We examined the effects of three different Wnt modulators on osteogenic differentiation using mesenchymal precursor cells (MPCs). These cells are a subset of the heterogeneous bone marrow-derived mesenchymal stem cell population that are selected based on the expression of the cell-surface antigens Stro-1 and CD106 (VCAM-1) [19], [20]. The use of such a defined subset has advantages when elucidating the role of signaling mechanisms within a cell population, as there is less scope for findings to be lost amongst a heterogeneous response from the mixed cell population. In addition, the proven beneficial properties of MPCs as compared to unselected MSCs [21] provides greater promise for their translation to the clinic.

Of the three small molecules tested in this study, the first, and our only agonist, is CHIR99021 (CHIR hereafter), a highly specific GSK3β inhibitor which activates canonical Wnt signaling [22]. The second and third are antagonists, being IWR-1, which inhibits canonical Wnt activity through its ability to stabilise Axin and the β-catenin destruction complex [23], and IWP-4, which is stated to inhibit the activity of both the canonical and non-canonical signaling pathways, by blocking all Wnt protein secretion [23]. By utilising these small molecules within our MBA platform, we were able to efficiently, and in a high throughout manner, screen for the effects of these molecules (or combinations thereof) on proliferation and in promoting or inhibiting MPC osteogenesis, through readout of the early osteogenesis marker alkaline phosphatase. Additionally, this screen allowed for the investigation of paracrine signaling effects that may be involved in osteogenesis, effects that would otherwise not be identified using conventional culture techniques alone. As well as providing insights into Wnt signaling activity in MPCs, this study shows the utility of such methods for the rapid screening of conditions that can be used to optimize cellular outputs for clinical applications. In particular, when combined with the use of small molecules, this methodology has significant potential to be applied in large-scale bioprocessing methods to tailor media compositions and ultimately replace more expensive cytokines.

## Materials and Methods

### Materials

All reagents were obtained from Gibco unless otherwise mentioned. CHIR99021 and IWP-4 were from Stemgent; IWR-1 was from Sigma-Aldrich.

### MPC Isolation and Culture

STRO-1-positive, human bone marrow-derived MPCs (Batches# M112 and M117) were prepared by Lonza (Walkersville, MD, USA) for Mesoblast Ltd (Melbourne, Australia), according to the procedure described by Gronthos et al [21], [24] and used under approval from the Medical Research Ethics Committee at the University of Queensland (#2010001069). These cells represent a fraction of the heterogeneous population of MSCs that are commonly isolated based on plastic-adherence alone. This MPC sub-population has been shown to contain the most potent stem cells, with properties that are advantageous to unselected MSCs [19], [20], [25] but may also provide a more consistent cellular response than would be expected when using cells from an unselected and more heterogeneous MSC population.

MPCs were cultured in αMEM supplemented with 100 U/ml penicillin, 100 µg/ml streptomycin (ps), 100 µM L-ascorbate-2-phosphate, 100 µM Sodium pyruvate and 10% batch-tested foetal bovine serum (FBS) at 37°C in 5% CO_2_ in an atmosphere with 95% humidity and passaged upon reaching 80% confluence. MPCs were characterized by their expression of the cell surface markers CD29 (BD Biosciences, Australia), CD44 (Invitrogen, Victoria, Australia), CD90 (R&D Systems New South Wales, Australia), CD146 (Invitrogen, Victoria, Australia), CD166 (Invitrogen, Victoria, Australia) and STRO-1 (kindly supplied to us by Prof. Stan Gronthos, Mesenchymal Stem Cell Group and Regenerative Medicine Program, Department of Haematology, SA Pathology; Co-Director, Centre for Stem Cell Research, Robinson Institute, University of Adelaide, Australia), and negative expression of the hematopoietic marker CD34 (Invitrogen, Victoria, Australia), and were shown to be capable of differentiation along the osteo-, chondro-, and adipogenic lineages **(Fig. S1)**.

### Microbioreactor Array Fabrication

MBA designs (described previously [8]) were drafted in AutoCAD software (Autodesk) and photoplotted onto HY2 glass plates (Konica Minolta, New South Wales, Australia). 100 µm high features were fabricated on silicon wafers using SU-8 2100 (MicroChem, Victoria, Australia) photolithography. Optical surface profilometry (Veeco NT1100, Plainview, NY) was used to confirm the feature heights and surface topography. Microbioreactor arrays were then fabricated using standard soft lithography with poly(dimethylsiloxane) (PDMS; Sylgard 184, Dow Corning, Midland, MI) [26]. To facilitate the easy removal of the PDMS mould, the SU-8 design features were first silanised with chlorotrimethylsiloxane (CTMS; Sigma Aldrich, Sydney). The bottom PDMS layer was bonded to a clean glass slide (100×76 mm, Proscitech, Thuringowa, Australia) using oxygen plasma (Harrick Plasma, 30 s, 10 W, 380 mTorr O_2_), and then the top PDMS layer was plasma-treated and aligned with the punched via holes in the bottom layer and sealed. The microbioreactors were then placed in an 80°C oven for several hours before sterilisation. Details of the MBA design and previous validation are reproduced in **Fig. S2**.

### Microbioreactor Array Culture and Analysis

Arrays were sterilised using an autoclave (121°C, 20 min), then vacuum-filled with sterile PBS containing 1% v/v Antibiotic-Antimycotic (A/A) using the channel outgas technique [27]. MPCs cultured in T175 flasks were harvested by incubation with Collagenase II for 30 min, followed by the addition of TrypLE Express to yield a suspension of single cells. Trypsin activity was neutralised with complete medium, then cells were counted and resuspended in complete medium at 5×10^6^ cells/mL. Using a 1 ml sterile syringe (Terumo) and sterilised blunt needle, cells were loaded into arrays in a single injection without introducing air bubbles. The inlet and outlet ports were plugged and arrays were placed in a sterile petri dish, then cells were allowed to attach for 3–4 hours. Tubing (PE50, 0.58 mm ID, BD Biosciences) of uniform length was cut, and to one end sterile blunt needles (22 gauge) were fitted and to the other end 22 gauge stainless steel needle tips were inserted, then the assembly was sterilized using 70% ethanol and dried using an oven (60°C).

Factor A, B, and C stock solutions (as indicated for each experiment) were diluted in osteogenic medium and drawn into syringes (1 mL, Terumo), attached to the tubing assembly and plugged into the MBA factor inlet ports A1, B1 and C1 respectively. Fresh osteogenic medium (Buffer A, B and C) was taken in another set of 3 syringes and plugged into the buffer inlet ports A0, B0 and C0. The syringes were placed on a syringe pump (NE-1800, New Era, Farmingdale, NY) and continuous fluid flow initiated at 36 µL/h total flowrate. The sterile petri dish housing the MBA was placed in the incubator, with tubes leading to the syringe pump that was placed outside the incubator at room temperature. The syringes were also covered with aluminium foil to reduce degradation of medium components by fluorescent room lights. MBA experiments ran for 6.5–7 d after the start of continuous fluid flow. Plate controls were also set up in parallel in 24-well plates (BD Biosciences), at identical surface densities, with the medium exchange occurring at the start of MBA fluid flow, and every 2nd day thereafter.

### Microbioreactor Array Endpoint Analysis and Imaging

At the experiment endpoint (7 d), arrays were washed once with PBS and then fixed/permeabilised with ice cold, 70% v/v ethanol for 15 min, then washed once more with PBS. An ELF97 Endogenous Phosphatase Detection Kit (Molecular Probes) was used to detect alkaline phosphatase activity, according to the manufacturer’s instructions. ELF97 working solution was applied until a yellow/green precipitate was observed (typically within 1–2 min), then phosphatase activity was stopped with 3 MBA volumes of PBS (pH 8.0), 25 mM EDTA and 5 mM tetramisole (Sigma), and washed finally with PBS. DNA was detected with 2 µg/mL propidium iodide and 100 µg/mL ribonuclease A. The microbioreactor was then washed 3 times with PBS before imaging. The same procedure was followed for static plate controls. 16-bit, multi-colour montage images of entire MBAs were imaged using a Zeiss LSM 710 laser scanning confocal microscope system and Zen 2008 acquisition software (Carl Zeiss). To compensate for intensity variations in the z-direction, 3 optical sections were acquired and then processed into a maximum intensity projection for image analysis. Images were linearly adjusted for publication. Static plate controls were imaged with an Olympus IX81 inverted fluorescence microscope, and ELF97 was detected using a DAPI longpass filter.

### Conditioned Medium Preparation

For experiments using conditioned media, media were collected at day 4 and 7 from MPCs grown in 6-well plates or T175 flasks, at half the nominal medium volume, both from cells cultured in growth conditions (growth-conditioned medium, GCM) and osteogenic differentiation conditions (osteo-conditioned medium, OCM). Media from both days were mixed and stored at 4°C until use, typically within a few days.

### RT-qPCR

Total RNA was extracted using the RNeasy Minikit with on-column DNase treatment (Qiagen) according to the manufacturer’s instructions. cDNA was synthesized from 1 µg RNA using 200 U SuperScript III, or the equivalent volume of DNase and RNase-free water for no-RT controls, in a total volume of 25 µl. qPCR reactions were set-up in a total volume of 10 µl with 1× Platinum SYBR Green qPCR SuperMix-UDG (Invitrogen) and 0.2 µM forward and reverse primers (Table 1). A 7500 Fast Real-Time PCR System (Applied Biosystems) with fast cycling parameters of 2 min at 50°C, 2 min at 95°C then 40 cycles of 3 sec at 95°C and 30 sec at 60°C followed by a melt curve was used to run the samples. Data were analysed using the 2^−ΔΔct^ method.

**Table 1:** qPCR Primer Sequences.

Marker	Gene	Primer	NCBI	Forward primer 5′-3′	Reverse primer 5′-3′	Ref
	Symbol	Bank ID	Accession #			
GAPDH	*GAPDH*		NM_002046	ATGGGGAAGGTGAAGGTCG	TAAAAGCAGCCCTGGTGACC	[39]
Axin 2	*AXIN2*	195927058c2		TACACTCCTTATTGGGCGATCA	AAGTTCGGAACAGGTAAGCAC	
β-catenin	*CTNNB1*			TGCCAT TCCACGACTAGTTCAG	CGTACG GCGCTGGGTATC	[40]
Dickkopf 1Homolog	*DKK1*		NM_012242.2	GGAAGCGCCGAAAACGCTGC	TCTGGAATACCCATCCAAGGTGCT	
Glycogen SynthaseKinase 3 Beta	*GSK3B*			AACTGCCCGACTAACAACAC	ATTGGTCTGTCCACGGTCTC	[41]
AlkalinePhosphatase	*ALPL*		NM_000478	GGGAACGAGGTCACCTCCAT	TGGTCACAATGCCCACAGAT	[42]
Runt-RelatedTranscriptionFactor 2	*RUNX2*		NM_001024630	AGTGATTTAGGGCGCATTCCT	GGAGGGCCGTGGGTTCT	[43]
Collagen Type 1Alpha 1	*COL1A1*		NM_000088	CCTGCGTGTACCCCACTCA	ACCAGACATGCCTCTTGTCCTT	[42]
Osteocalcin	*BGLAP*		NM_199173	AGCAAAGGTGCAGCCTTTGT	GCGCCTGGGTCTCTTCACT	[42]
Osteonectin	*SPARC*		BC008011	CCTGGATCTTCTTTCTCCTTTGC	ATCAGGCAGGGCTTCTTGCT	[42]
Osteopontin	*SPP1*		BC022844	ACCTGAACGCGCCTTCTG	CATCCAGCTGACTCGTTTCATAA	[42]
Msh homeobox 2	*MSX2*	84452153c1		ATGGCTTCTCCGTCCAAAGG	TCGTCGGGCGAAAACAAGTC	
Distal-lesshomeobox 5	*DLX5*			GACTTCCAAGCTCCGTTCCA	CTGTAGTAGTCAGAATCGGTAGCTGAA	[44]
Cyclin D1	*CCND1*		NM_053056	CCCTCGGTGTCCTACTTCAA	AGGAAGCGGTCCAGGTAGTT	[45]

### pNPP Assay

MSCs were cultured for 7 d in osteogenic medium supplemented with varying concentrations of CHIR. After 7 days the samples were lysed in 150 µl 0.1% Triton-X-100 in 0.2 M carbonate buffer and subjected to 3 freeze-thaw cycles between −80°C and 37°C. To determine alkaline phosphatase activity, 50 µl working substrate (0.3 mg/ml pNPP (Sigma) and 3.3 mM MgCl_2_ in 0.2 M carbonate buffer) was added to each sample and incubated at 37°C before measurement of the absorbance on a Spectramax M5 Fluorometer (Molecular Devices) with an excitation wavelength of 405 nm. pNPP concentration was determined by extrapolation form a standard curve and normalized to both incubation time and DNA content as assessed by PicoGreen assay (Molecular Probes, performed according to the manufacturer’s instructions).

### Data Analysis and Statistical Methods

MBA data analysis proceeded as previously [8]. Briefly, total fluorescence intensities (*T_ELF97_*, for example) were extracted from array images with AGScan software (Sigenae; http://www.sigenae.org). Expression indices were derived by linearly transforming spot intensities in each channel about the mean and standard deviation for all spots in an individual array, by *I_ELF97_* = (*T_ELF97_−µ_ELF97_)*/*σ_ELF97_*, where *I_ELF97_* is termed the expression index of ELF97, and *µ*_E*LF97*_ is the mean and *σ*_E*LF97*_ the standard deviation of all spot intensities (*T*_E*LF97*_). Heat maps were generated with MATLAB software (The MathWorks). Factorial analyses were performed on expression indices with MINITAB 15 software (Minitab Inc.). *p*-values for factorial analysis were calculated by MINITAB after analysing the general full-factorial design for 2 replicate arrays each of 2 donors, and including factor effects up to the third order. Pearson’s correlation coefficients (*r_X,Y_*) were calculated with Microsoft Excel. For pair wise comparisons, one-way ANOVA with post-hoc Tukey or Games-Howell tests were performed with SPSS Statistics 20.0, and differences with *p*<0.05 were considered significant. Kolmogorov-Smirnov tests were used for data normality, and Levene’s tests for homogeneity of variance. EC50 measurements were determined using GraphPad Prism software (version 6.00) to perform non-linear regression and log (agonist) vs. response-Variable slope (four parameters) tests.

## Results

### Validation of Microbioreactor Array Culture Parameters for MPC Seeding and Osteogenesis

We first identified MBA culture parameters most conducive to MPC culture and osteogenic differentiation, by varying culture chamber heights (100 and 250 µm), medium perfusion rates (6.2 and 10.3 µL/h/cm^2^), and culture substrates (glass, FBS, collagen I). In all cases, these conditions were evaluated over a 7 day culture period to match the later osteogenic assays.

### MBA Culture Chamber Size, Substrate Coating, and Medium Flowrate

**Figure 1:**
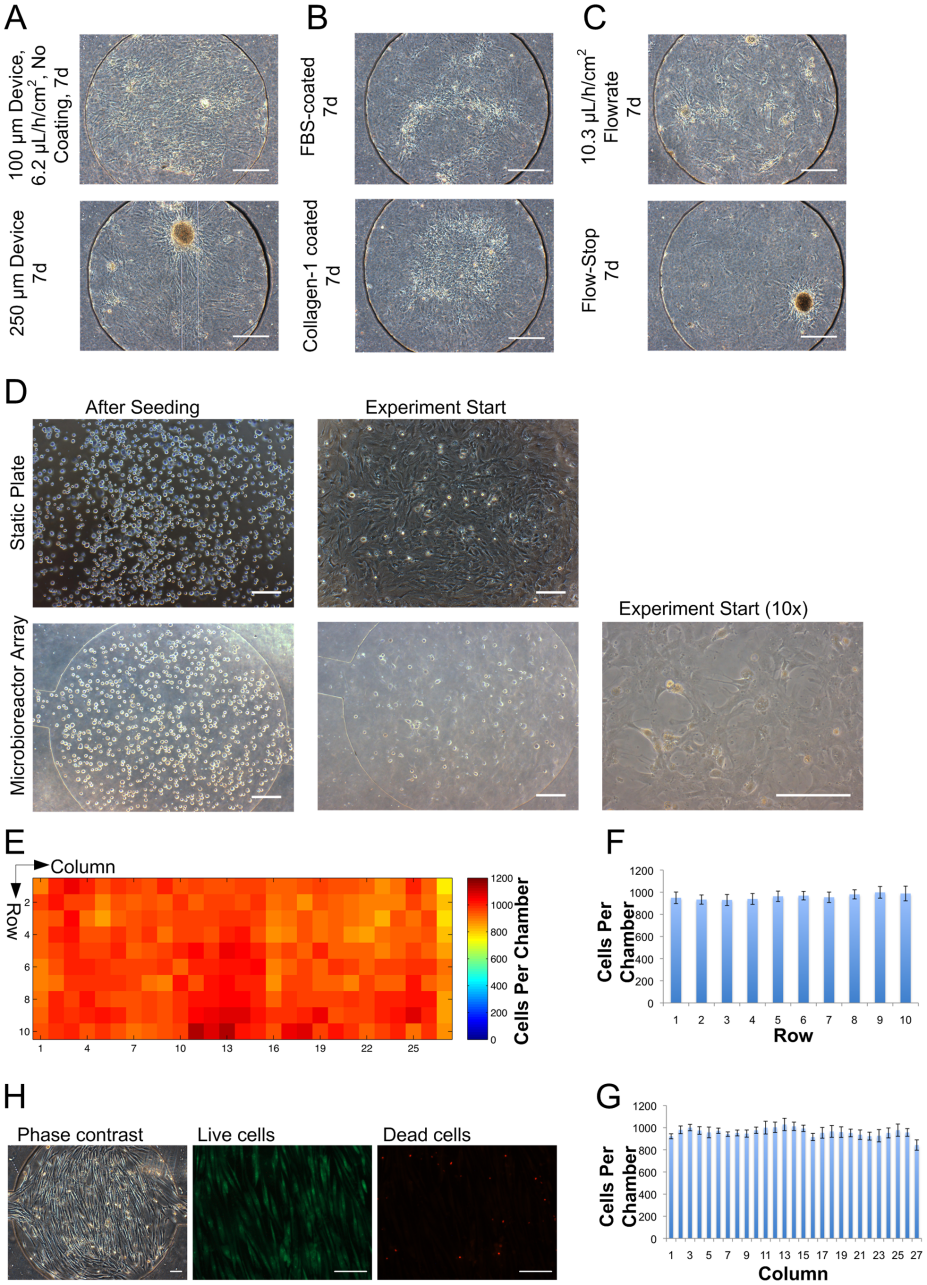
Validation of MBA culture parameters and MPC seeding. A Comparison of cell morphology in 100 µm (top) versus 250 µm-high (bottom) devices. Scale bar, 200 µm. B Comparison of medium exchange regimes varied from conditions in top panel of A –10 µL/h flowrate (top) and periodic flow-stop (bottom). Scale bar, 200 µm. C Comparison of surface coating regimes varied from conditions in top panel of A – FBS-coated substrate (top) and collagen-I-coated substrate (bottom). Scale bar, 200 µm. D Phase contrast micrographs of MPCs in static plate controls and microbioreactor arrays in suspension directly after seeding, and attached after 4–5 h, just prior to the start of fluid flow. Scale bar, 200 µm. E Heatmap showing distribution of MPCs seeded into a MBA at representative experimental densities. F Graph showing average cells per chamber as a function of row. G Graph showing average cells per chamber as a function of column. H Live/dead staining of MPCs after 7 days. Scale bar, 100 µm.

MBAs fabricated to 100 µm feature height produced cell cultures with a homogeneous monolayer appearance after 7 days of differentiation, whereas cells in the 250 µm MBAs were more susceptible to aggregation into 3D structures, which were unsuitable for screening purposes **(** **Fig. 1A** **)**. Coating of the glass substrate with either FBS or Collagen-I prior to cell seeding was also tested to determine whether this would enhance cell attachment or morphology. These were found not to have any noticeable enhancing effects and so were not adopted for subsequent experiments **(** **Fig. 1B** **)**. Finally, varying medium perfusion regimes were tested. A 6.2 µL/h/cm^2^ (36 µL/h total) flowrate performed better than 10.3 µL/h/cm^2^ or a flow-stop medium exchange regime (**Fig. 1C**), as assessed by the maintenance of a single monolayer of cells with minimal aggregation. As a result of this optimization, all further experiments were performed using a feature height of 100 µm and flow rate of 6.2 µL/h/cm^2^, without prior coating of the bioreactor substrate. The physical parameters of the MBA operation under nominal conditions are given in **Table S1**.

### Cell Seeding Distribution

Given the importance of initial cell density on mesenchymal stem cell differentiation [28], we also wanted to confirm that the seeding strategy provided a confluent monolayer of MPCs, with an equal distribution throughout the chambers of the MBA. At the initiation of medium perfusion four hours after cell seeding, MPCs seeded at a target density of 50,000 cells/cm^2^ had formed a confluent monolayer. The degree of cell spreading and confluency was similar for MPCs in the MBA and those in static plate controls **(** **Fig. 1D** **)** and was deemed suitable for the investigation of osteogenic differentiation. To demonstrate that the distribution of MPCs throughout the various chambers of the array was homogeneous, MPCs were fixed, labeled with Hoechst, then injected into the array. The array was imaged, and nuclei quantified by image analysis. Cells were uniformly distributed throughout the array **(** **Fig. 1E–G** **)** with an average seeding density of 961±48.6 s.d. cells per chamber, equivalent to a surface density of 46 000±2330 s.d. cells/cm^2^ (coefficient of variation, 5.1%). Post cell seeding and culture, live/dead staining was performed to ensure the viability of MPCs within the MBA. This showed good viability of the MPC population after 7 days under continuous medium perfusion in the MBA **(** **Fig. 1H** **)**.

This thorough optimization of the MBA parameters and seeding protocol ensured good compatibility of MPCs in subsequent molecular screens.

### Microbioreactor Array Screening of the Effects of Wnt Modulators on MPC Osteogenesis

**Figure 2:**
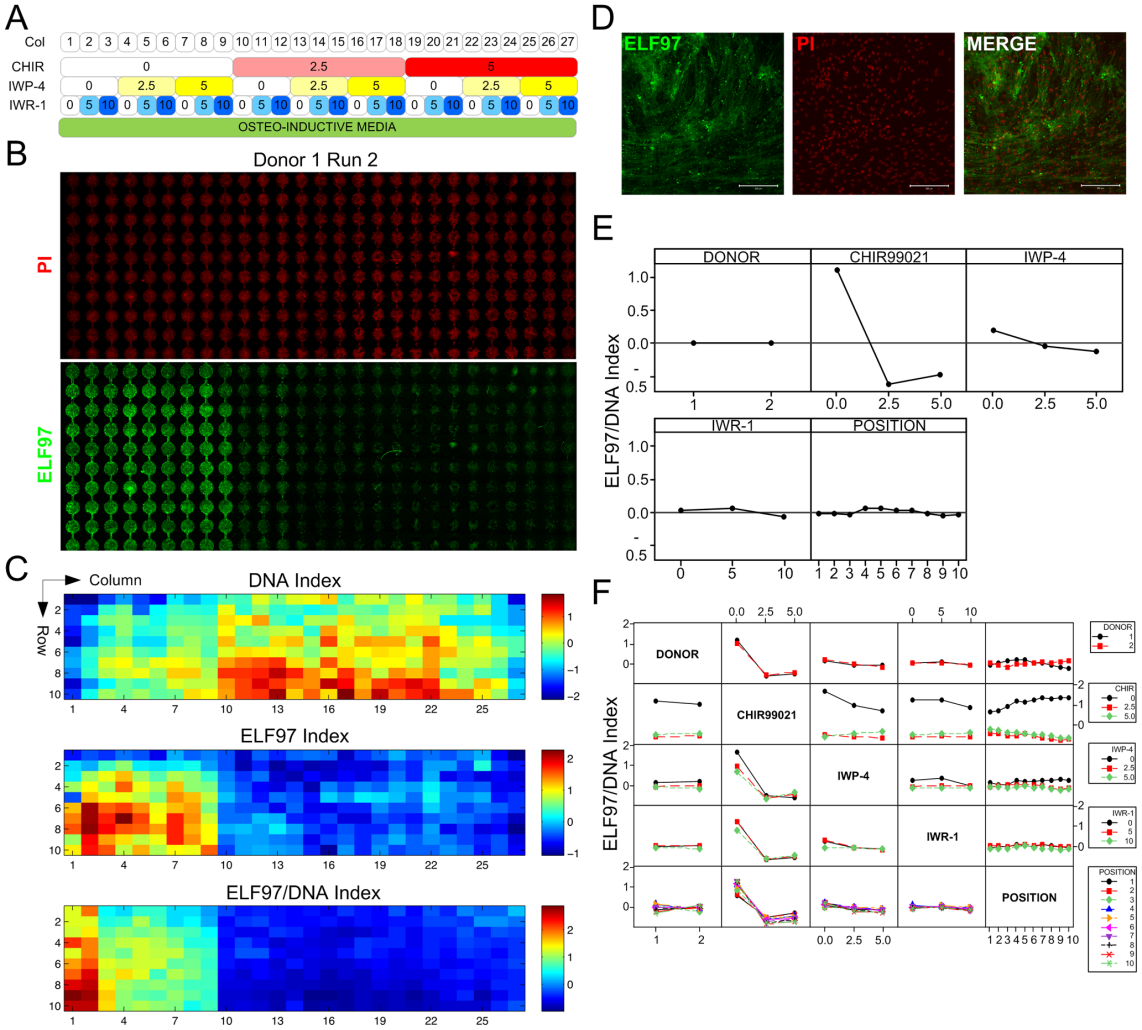
MBA screening of Wnt modulators in MPC osteogenesis. A Panel of screening conditions in MBAs. Numbers denote concentrations of the various molecules, in µM. B Confocal microscopy images of endpoint PI (DNA) and ELF97 (alkaline phosphatase activity) staining from a representative experiment. Direction of fluid flow was from top to bottom. C Heatmaps of expression indices (see Methods) for DNA, ELF97, and ELF97/DNA ratio. The average expression index of 2 runs from each of 2 MPC donors (4 in total) is shown, and units represent global standard deviations of difference relative to the global mean. For data from individual runs, see Figs. S2–S5. D Higher magnification fluorescence images of representative MPCs in MBA displaying alkaline phosphatase activity (ELF97) and DNA staining (PI). Scale bar: 200 µm. E Main effects plot showing effect of DONOR, CHIR99021 (CHIR), IWP-4, IWR-1 and POSITION on expression index for ELF97/DNA ratio. F Interaction effects plot showing effects of 2 combined factors on ELF97/DNA ratio.

Using the validated MBA conditions, MPCs were screened with osteogenic medium supplemented with combinations of the Wnt modulators, CHIR, IWR-1 and IWP-4, which act as an agonist of canonical Wnt, an antagonist of canonical Wnt and an antagonist of both canonical and non-canonical Wnt signaling respectively. The MBA screening results in application of a full-factorial array of three concentrations each of the three factors, each with differing effects on Wnt pathway activity. Within each condition the medium flows through a column of 10 serially-connected culture chambers. The compositions formed in the various columns of the array are given in **Fig. 2A**.

### MBA Screen Performance

After 6.5–7 days of culture under continuous slow perfusion of the various conditions, the arrays were fixed and analysed *in situ* for alkaline phosphatase activity (using an ELF97 endogenous phosphatase detection kit) as a marker for early osteogenic differentiation, and nuclear DNA staining (propidium iodide, with RNase digestion) as a surrogate measure of cell number, a representative example of which is given in **Fig. 2B,D** **.** Experiments were performed to gain data for MPCs from two different donors with two independent experiments for each, and fluorescence levels of ELF97, DNA, as well as the DNA-normalised level of ELF97 (ELF97/DNA) are reported for each chamber in the MBA. Individual results from each run are shown in **Figures S3–S6,** and pooled data from all four runs is summarized in **Fig. 2C**. Data for each of the metrics (ELF97, DNA, ELF97/DNA) were highly correlated between the four runs, having Pearson’s correlation coefficients for paired chambers between runs of 0.3–0.81, with the main metric of interest, ELF97/DNA ranging from 0.58–0.81 **(** **Table 2** **)**. This is also highlighted by a heat map comparison of the different runs **(Fig. S6)**.

**Table 2:** Pearson’s correlation coefficients for paired chambers from individual MBA experiments.

Donor/Run	Donor 1 Run 1			Donor 1 Run 2			Donor 2 Run 1		
	DNA	ELF97	ELF97/DNA	DNA	ELF97	ELF97/DNA	DNA	ELF97	ELF97/DNA
**Donor 1 Run 2**	0.37	0.31	0.67						
**Donor 2 Run 1**	0.41	0.44	0.76						
**Donor 2 Run 2**	0.4	0.45	0.58	0.23	0.39	0.59	0.3	0.52	0.68

### MBA Wnt Modulator Screening Results

The screening results showed strong ELF97 staining for MPCs treated with osteogenic medium alone (**Fig. 2A–C**, Column 1), which confirmed the expression and activity of alkaline phosphatase, and the successful induction of osteogenic differentiation under array conditions. Factorial analysis was then performed using data from all of the 4 runs (**Fig. S8**), to estimate the effect magnitude (**Fig. 2E, F**) and significance (**Table 3**) of individual and combined factors. This showed that there were statistically significant effects (**Table 3**) for each of the individual compounds, as well as significant interaction effects between CHIR and both IWR-1 and IWP-4. This data also confirmed that both the donor and the replicate experiments of each donor were not significant sources of variation in the data (**Table 3**). Together, these provided a high degree of confidence in the quality and reproducibility of data from the MBA screen, with significant effects of the Wnt-modulatory compounds which could then be probed further to elucidate their effects upon osteogenesis.

**Table 3:** Significance of factor effects from factorial analysis.

Source of Variance	DNA Index p-value	ELF97 Index p-value	ELF97/DNA Index p-value
Blocks	1	1	1
DONOR	1	1	1
CHIR99021	0	0	0
IWP-4	0.29	0.004	0
IWR-1	0.019	0.003	0.002
ROW	0	0	0.629
DONOR*CHIR99021	0	0.312	0.003
DONOR*IWP-4	0	0	0.135
DONOR*IWR-1	0.828	0.877	0.648
DONOR*ROW	0.003	0.021	0
CHIR99021*IWP-4	0	0.1	0
CHIR99021*IWR-1	0	0.429	0
CHIR99021*ROW	0	0	0
IWP-4*IWR-1	0	0.001	0
IWP-4*ROW	0.538	0.618	0.379
IWR-1*ROW	0.98	0.929	1
DONOR*CHIR99021*IWP-4	0.379	0	0
DONOR*CHIR99021*IWR-1	0.854	0.084	0.007
DONOR*CHIR99021*ROW	0	0.003	0
DONOR*IWP-4*IWR-1	0	0.254	0.043
DONOR*IWP-4*ROW	0.858	0.933	0.997
DONOR*IWR-1*ROW	0.623	0.845	0.997
CHIR99021*IWP-4*IWR-1	0.125	0.005	0
CHIR99021*IWP-4*ROW	0.773	0.839	0.155
CHIR99021*IWR-1*ROW	0.996	1	0.999
IWP-4*IWR-1*ROW	0.981	1	1

In the absence of any other factors, increasing concentrations of IWR-1 resulted in a decrease in the alkaline phosphatase activity per cell (i.e. the ELF97/DNA ratio). The highest concentration of IWR-1 (10 µM) clearly inhibited osteogenesis (**Fig. 2C** **, column 3**), however, the ELF97/DNA ratio at an intermediate concentration of IWR-1 (5 µM) was similar overall to that observed for MPCs cultured in osteogenic medium alone, suggesting that this concentration was below that required to sufficiently suppress the canonical Wnt pathway (**Fig. 2C** **, column 2**). The results of treatment with IWP-4 alone (**Fig. 2C** **, column 4 (2.5 µM)) and column 7 (5 µM)**) confirmed that it was mildly inhibitory to osteogenesis, with clear reductions in the ELF97/DNA activity compared to the osteogenic media alone, and no significant effects of concentration. However, the most striking result from the MBA screen was the clear reduction in ELF97 staining, evident in all wells that had been treated with CHIR, regardless of the concentration or the combination of other factors provided **(** **Fig. 2C** **, columns 10–27;** **Fig. 2E,F** **)**. This was correlated with an increase in DNA content, particularly for culture chambers towards the end of the column, leading ultimately to a large reduction in the alkaline phosphatase activity per cell (ELF97/DNA ratio).

In the absence of CHIR, combinations of IWR-1 and IWP-4 showed different patterns of activity to the individual factors. With these factors in combination, IWP-4 caused a concentration-dependent decrease in the ELF97/DNA signal as the concentration of IWR-1 increased. There were no changes in DNA content across these conditions, showing that the effect was solely attributed to changes in the alkaline phosphatase activity between the culture conditions (**Fig. 2C** **, columns 1–9**). The over-riding inhibitory effect of CHIR to diminish osteogenesis meant that no clear differences could be determined between any of the conditions in which CHIR was included.

### Validation and Further Investigation of MBA Screening Outcomes in Static Culture

**Figure 3:**
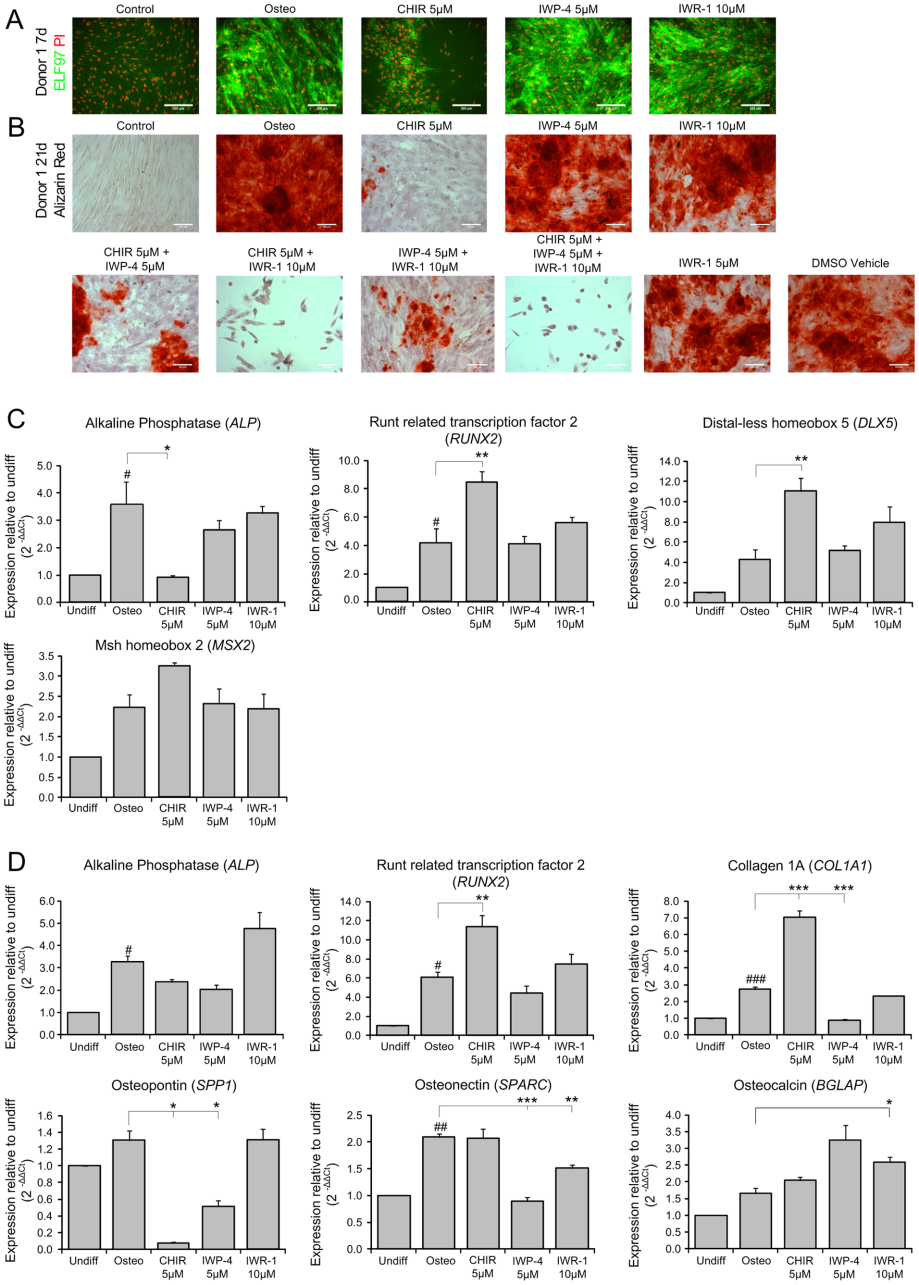
Analysis of selected inhibitor concentrations on osteogenesis under standard conditions. A ELF97 (green) and PI (red) staining of MPCs treated with CHIR, IWP-4 and IWR-1 for 7 days. Scale bar, 100 µm. B Alizarin red staining of MPCs treated with combinations of CHIR, IWP-4 and IWR-1 for 21 days. Scale bar, 100 µm. C) RT-qPCR determination of expression of osteogenic marker genes after 7 days D) qPCR determination of expression of osteogenic markers genes after 21 days. RT-qPCR data is shown as mean±SEM. N = 3, p<0.05 (*), p<0.01 (**), p<0.001 (***).

To more closely investigate the underlying events responsible for the surprising osteogenic inhibition in the presence of *both* Wnt agonist and antagonists, we first confirmed that the results from the MBA screen were applicable to cells cultured in standard culture formats (static plates), prior to the use of these conditions for more conventional analysis techniques. ELF97 staining of static MPC cultures after 7 days treatment with 5 uM CHIR, 10 uM IWR-1 or 5 uM IWP-4 confirmed the primary results from arrays, showing an increase in ELF97 staining when MPCs were cultured with osteogenic supplements, which was strongly inhibited with the inclusion of CHIR **(** **Fig. 3A** **)**. A dose-response curve also confirmed that CHIR was profoundly inhibitory upon ALP activity at all concentrations above 1 µM **(Fig. S9)**.

### Effects on Late Osteogenesis Markers

We further investigated each molecule’s effects on late osteogenesis, using Alizarin red staining to determine the extent of mineral deposition after 21 days. These results mirrored those of the ELF97 staining, with osteogenic supplements inducing the formation of Alizarin red-positive deposits across the majority of the culture surface. This was almost completely abolished in the presence of CHIR and inhibited to a lesser extent by either IWP-4 or IWR-1 at the concentrations tested **(** **Fig. 3B** **)**. This confirmed that effects detected in the MBA and static plate, using 7 days ELF97 staining as an early readout, translated through to an equivalent influence on the final maturation of MPCs into mineralizing osteoblasts. Together these data provided confidence that we could use conventional cultures to further investigate the changes seen in the MBA screen.

### Modulation of Gene Expression

**Figure 4:**
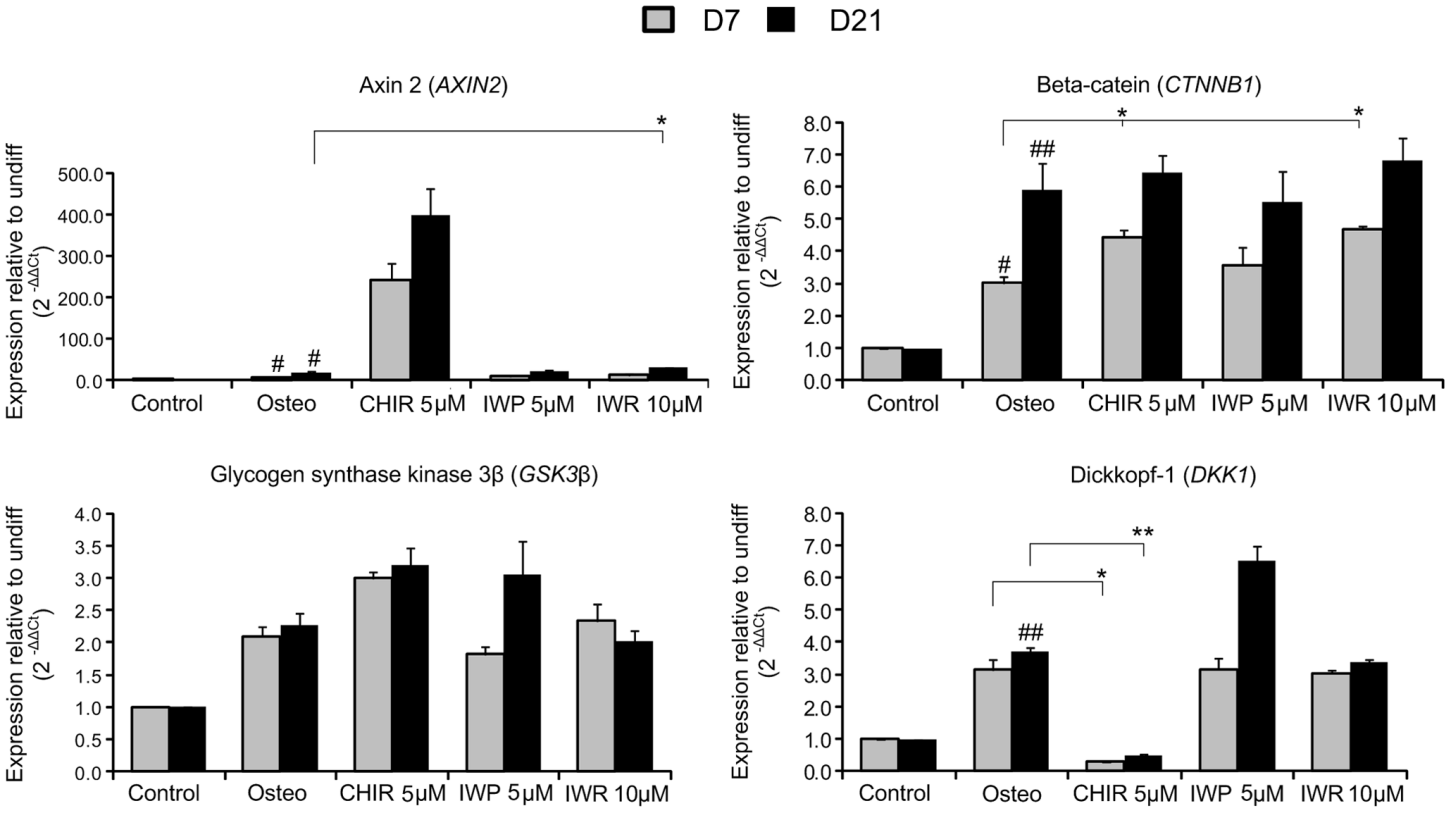
qPCR determination of the expression of Wnt related factors. qPCR determination of expression of Wnt pathway genes in MPCs after 7 and 21 days treatment. Data is shown as mean±SEM. N = 3, p<0.05 (*), p<0.01 (**), p<0.001 (***).

Using these static cultures, we then utilised RT-qPCR to measure any changes in the expression of a number of key members of the Wnt signaling pathway and determine how they were influenced by CHIR, IWR-1 and IWP-4 treatments. As would be expected due to its role as a canonical Wnt agonist, CHIR treatment of MPCs caused upregulation of *AXIN2* (regarded as a marker of canonical Wnt pathway activation, [29], [30]), as well as *CTNNB1 (*β-catenin) and *GSK3B,* whilst the Wnt inhibitor *DKK1* was downregulated at both 7 and 21 days **(** **Fig. 4** **)**. MPCs treated with IWP-4 and IWR-1 showed no significant changes in the expression of *AXIN2*, *CTNNB1* and *GSK3B* as compared to osteogenic medium alone on day 7, but MPCs treated with IWP-4 expressed elevated levels of *DKK1* and *GSK3B* on day 21. The significant upregulation (up to 350-fold) of *AXIN2* in CHIR-treated MPCs at both day 7 and 21 provided a strong indication that CHIR was working in the manner expected (to activate canonical Wnt signaling) and so we next analysed the expression of markers of different stages of osteogenesis to elucidate why CHIR may be acting to inhibit differentiation and what differences may be observed between the agonist CHIR, and antagonists IWR-1 and IWP-4.

Determination of gene expression at 7 days showed that the early osteogenic transcription factors *RUNX2*, *MSX2* and *DLX5* were significantly upregulated in MPCs treated with CHIR **(** **Fig. 3C** **)**. However, (correlating with the findings from the MBA screen) *ALP* expression was significantly inhibited by CHIR **(** **Fig. 3C** **)** Gene expression data for 21 day cultures showed that this upregulation of *RUNX2* and downregulation of *ALP* expression was maintained throughout differentiation. At this later timepoint, *SPP1* (Osteopontin) expression was also decreased, whilst *COL1A1* (Type-I-collagen) levels increased and no significant changes were observed for *SPARC* (Osteonectin) or *BGLAP* (Osteocalcin) expression **(** **Fig. 3D** **)**.

Consistent with the results from the MBA screen, the effects of IWP-4 and IWR-1 upon gene expression levels were weaker than that of CHIR. However, both IWR-1 and IWP-4 decreased expression levels of *ALP* without the simultaneous increase in *RUNX2*, *MSX2* and *DLX5* observed using CHIR **(** **Fig. 3C** **)**. After 21 days, *ALP* expression under IWR-1 treatment was similar to untreated controls but was still reduced with IWP-4 treatment. At this later timepoint, IWP-4 also caused a significant downregulation of *SPARC* and *COL1A1*, whilst only a significant reduction in *COL1A1* was observed using IWR-4 **(** **Fig. 3D** **)**.

### Involvement of Paracrine Factors in MPC Osteogenic Differentiation

**Figure 5:**
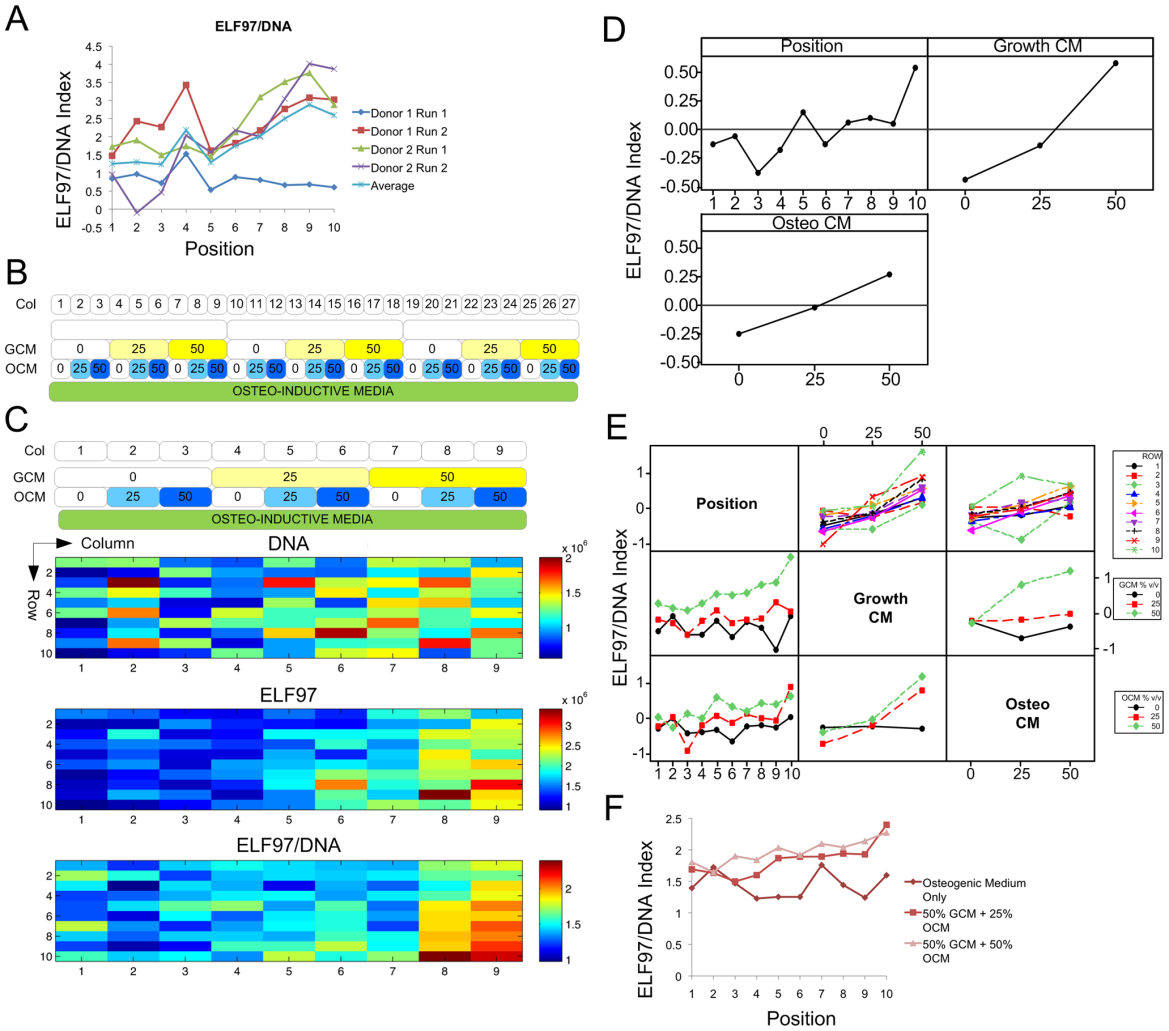
Screening MPC growth- and differentiation-conditioned medium in MBAs. A Traces of ELF97/DNA expression index against row, from column 1 of all microbioreactor runs from Figure 2 (pooled arrays), and the average value. B Panel of conditions formed in conditioned medium screening experiment. C Heatmaps of total expression intensities (arbitrary units) for DNA, ELF97, and ELF97/DNA ratio. The average response of 3 technical replicates from one experimental run is shown. D Main effects plot showing effect of ROW, Growth-conditioned medium and Osteo-conditioned medium on expression index for ELF97/DNA ratio. E Interaction effects plot showing effects of 2 combined factors on ELF97/DNA ratio. F Traces of ELF97/DNA expression index against row, from columns 1, 8 and 9 (average of 3 technical replicates).

A further finding from the MBA screen **(** **Fig. 2** **)**, was that in Column 1, which contained just osteogenic medium and no modulators, the peak absolute ELF97 and ELF97/DNA activity occurred not in the initial rows of the array, but further downstream **(** **Fig. 2C** **)**. This effect was more clearly shown in traces of ELF97/DNA Index versus Row coordinate for the microbioreactor runs, which revealed an increasing trend in ELF97/DNA activity in downstream rows, with the exception of Donor 1 Run 1 **(** **Fig. 5A** **)**. To confirm this effect, row-dependent alkaline phosphatase activity was further observed by Fast Blue staining of cells grown in an independent MBA experiment **(Fig. S10)**. Our previous work suggests this is a potential signature of a process influenced by paracrine factors [8]. This is based on the concept that downstream chambers received medium that has been conditioned more (i.e. factors and nutrients depleted, and paracrine factors and wastes supplemented) by upstream cells.

A similar paracrine effect was observed, but for DNA content, in columns treated with CHIR, suggesting that CHIR may induce MPCs to secrete a factor that enhances their proliferation.

### MBA Screening of Conditioned Medium Fractions

To more effectively confirm that paracrine factors were responsible for the enhancement in osteogenic differentiation in the lower rows of the MBA, conditioned medium was collected from cultures of both undifferentiated MPCs treated with growth medium (termed GCM), and MPCs undergoing osteogenesis under treatment with osteogenic medium (termed OCM) (see Methods). GCM and OCM were each titrated in at 0, 25, and 50% v/v, with medium otherwise composed of fresh osteo-inductive medium. Factor/buffer channel A was left blank, so one array run comprised 3 technical replicates **(** **Fig. 5B** **)**.

Application of the conditioned medium fractions appeared to enhance osteogenesis, corresponding both to an increase in the average level of ELF97/DNA activity in a whole column, as well as a shift in the higher intensities of ELF97/DNA expression towards the upstream rows in the array **(** **Fig. 5C** **)**.

As for the effects of increasing amounts of GCM and OCM fractions, the ELF97 index was slightly increased with 50% GCM, although there appeared to be no significant effect on ELF97/DNA index from either fraction when added individually up to 50% v/v. Taken as an average effect across all combinations however, both fractions appeared to increase the ELF97/DNA index **(** **Fig. 5D** **)**, and appeared to have an additive effect when combined at higher concentrations **(** **Fig. 5E** **)**. 50% GCM with either 25 or 50% OCM increased ELF97/DNA activity above the other conditions, and also appeared to shift the onset of high-level ELF97/DNA expression towards the upstream chambers in the array **(** **Fig. 5F** **)**. In these conditions, the medium comprises only 25 or 0% v/v fresh osteogenic medium, respectively, suggesting that the more conditioned medium that was present, there was a greater improvement in differentiation. 50% OCM +25% GCM resulted in a slightly increased ELF97 index, but a ELF97/DNA index comparable to the baseline, suggesting that any enhancing factors may be present at higher levels in the GCM. Attempts to translate this to macroscale culture were met with mixed results (data not shown), suggesting that it is a difficult phenomenon to control within a standard plate culture format.

## Discussion

In this study, we were able to successfully culture and differentiate MPCs for prolonged culture periods (up to 7 days) within our MBA. This facilitated combinatorial analysis of the impact of small molecule modulators of Wnt signaling upon the osteogenic differentiation of MPCs. In addition to the observations outlined here regarding Wnt signaling, this work opens opportunities for future applications in which the ability to perform cell-based assays with MPCs within such a platform may be used to examine a wide range of microenvironmental conditions and cellular signaling pathways, as well as differentiation of MPCs into a number of lineages. Importantly, these future applications will benefit broadly from the precisely controlled microenvironmental conditions, ease of multiplexing, and reduction in reagent usage inherent in such a platform.

By optimizing the conditions of the MBA, we were able to validate a combination of culture parameters, in terms of culture chamber height, medium perfusion rate and culture substrate, that resulted in MPCs, which were viable for the 7 day period required for our assay and also able to undergo osteogenic differentiation. Furthermore, we confirmed that cells were evenly distributed throughout the bioreactor and maintained as a homogenous monolayer- both criteria that are vital in applications where image analysis is used to provided an accurate quantitative readout.

As a part of this optimisation process, the exchange rate of the culture medium was selected to ensure cell viability whilst providing the least cellular aggregation. Medium flowrate is known to influence several phenomena: fluid shear force, the diffusive/convective balance of supply of nutrients and factors, and removal of metabolic wastes and secreted factors and so the effect of medium flow in our system was considered. Previous in vitro studies using 2-D macroscale models have shown that shear stress does have a significant on osteogenesis but have found that it needs to be in the range of 0.1–0.5 Pa, with a few studies showing stress values as high as 2 Pa are required [31]. These studies correlate well with the shear stress levels that are expected to occur *in vivo* within bone (0.8–3 Pa) [31]. In this current work, the medium flowrate and geometry used results in very low shear stress (1.36×10^−4^ Pa) – much smaller than values which have been used to influence MSC osteogenic differentiation [32], [33] – therefore the impact of shear stress upon the cellular behaviours observed was thought to be minimal compared to the effects of the factor treatments.

Further to the parameter optimization, data from the MBA showed a high degree of consistency between both independent MPC donors and runs. This correlation between runs and donors was at levels comparable to previous work with hESCs in MBAs, which had correlation coefficients of 0.66–0.69 for a single cell line [8] and provided a high degree of confidence in both the quality and reproducibility of the data obtained from the MBA.

Data from the MBA screening showed that all three Wnt modulatory compounds influence osteogenic activity and therefore correlate with previous reports that suggest that Wnt signaling has an important role to play in osteogenesis. However, our results were somewhat surprising given that stimulation of the canonical Wnt pathway (with CHIR)had inhibitory effects upon osteogenesis, confounded by our observation that inhibition of both canonical, or total (canonical and non-canonical) Wnt activity (via 10 µm IWR-1 and 2.5–5 µm IWP-4, respectively) also inhibited osteogenesis, albeit to a lesser extent.

Previous reports have suggested that canonical Wnt activity can stimulate osteogenesis, although these studies did not necessarily use primary hMSCs and had used varying means to enhance canonical Wnt activity [14], [15], [34]. Furthermore, there is evidence to suggest that non-canonical Wnts may enhance osteogenesis [17]. The suggestion of a pro-osteogenic effect of Wnt signaling from these studies align well with our findings that high concentrations of both IWR-1 and IWP-4 (Wnt antagonists) reduced both the ELF97/DNA index in the MBA screen and decreased the expression level of key osteogenic marker genes in subsequent static cultures. Interestingly, the stronger effect of IWP-4, as compared to IWR-1 (which required a higher concentration to effect any changes in the ELF97/DNA index), fits well with the fact that IWP-4 inhibits *all* Wnt signaling the effects of IWR-1 is restricted purely to canonical mechanisms, supporting the hypothesis that both canonical and non-canonical Wnt activity has a role to play in enhancing osteogenic outcomes.

The primary finding that CHIR also inhibited osteogenesis (and to a much greater extent than either IWR-1 or IWP-4) was unexpected due to the previously noted role of such signaling to enhance osteogenesis [15], [16]. This inhibitory action of CHIR was also particularly surprising in light of **t**he significant upregulation of both Wnt signaling molecules *(CTNNB1 (*β-catenin), *GSK3β and AXIN2,* which is commonly regarded as a marker of canonical Wnt pathway activation, [29], [30]) as well as upregulation of the pro-osteogenic transcription factors *RUNX2*, *MSX2* and *DLX5* at Day 7 in MPCs treated with CHIR. These changes in gene expression were consistent with both with the activity of CHIR as a canonical Wnt agonist and the expectation that Wnt signaling would increase osteogenesis.

Conversely, the observed down-regulation of *ALP* was contradictory to previous data showing that canonical Wnt signaling promotes *ALP* expression [34]. One explanation for these results may be the use of Dexamethasone (Dex) as an osteogenic agent; canonical Wnt signaling (induced by either Wnt3a or LiCl) has previously been shown to decrease both *ALP* and mineralization and increase hMSC proliferation in the presence of Dex [13]. However, in experiments performed in the absence of Dex, another, less specific small molecule inhibitor of GSK3β (BIO) was shown to enhance osteogenesis [35]. In the absence of CHIR, Dex is known to induce the expression of *ALP* via the activity of an as yet unidentified intermediate protein [36], thereby raising the possibility that the effect of CHIR upon *ALP* is mediated via this factor.

Interestingly, our results also showed that although the pattern of high *RUNX2* and low *ALP* was maintained in cultures after 21 days and resulted in a reduction in *SPP1* expression, *COL1A1* expression was elevated. This may indicate different pathways leading from Wnt activity through to the expression of *SPP1* and *COL1A1*. *ALP* has been linked to *SPP1* expression (where it is hypothesized that the generation of free phosphate by alkaline phosphatase may act to induce SPP expression [37], [38]) and so it may be that inhibition of *ALP* by CHIR reduces *SPP1* expression and subsequent maturation, whilst *COL1A1* expression is elevated by the enhanced Wnt activity but is not sufficient to ensure a mature osteogenic phenotype.

The second major finding from the MBA screen was the observation of differential effects along the columns of the bioreactor. We have previously observed similar effects when using the MBA and shown that they are caused by the paracrine effects of factors accumulating in the culture medium as it passes over the cells [8]. This data therefore suggested that factors secreted by the MPCs in the upstream chambers act to enhance differentiation in MPCs in the downstream chambers, a hypothesis is further supported by the observation that conditioned culture medium increased both the average ELF97/DNA activity as well as shifting higher ELF97/DNA intensities towards the upstream rows in the array.

The observation that GCM and OCM enhanced osteogenic differentiation in the arrays may suggest a threshold level of required paracrine factor accumulation in conditioned medium. This is supported by the fact that the more conditioned medium that was present, the better the outcome of differentiation. Greater enhancement was observed with the application of GCM, suggesting that the relevant paracrine factors are found in either GCM or OCM, but are perhaps more prevalent in the GCM fraction. This is an interesting finding, as it may explain why osteogenic differentiation in static cultures is critically dependent on the state of the culture at initiation of differentiation – the outcome may depend not only on the cell density, but also the *pre-culture time*, which affects production and binding of factors contained in GCM.

Such insights have important implications for cell processing procedures, as they highlight a microenvironmental culture parameter (paracrine factor accumulation) which impacts on differentiation outcomes, that can ultimately be regulated through macroscale process parameters (culture architecture, vessel design, and medium exchange rate). Although the MBA screening provides some indications and “hit” conditions, they must be followed up with appropriate macroscale experiments to confirm the impact of the putative effects.

## Conclusions

We have developed a consistent and reliable set of conditions for screening modulators of signalling activity in MPCs cultured under continuous perfusion in a MBA undergoing osteogenesis. Using Wnt signalling as a proof-of-concept system, this work clearly demonstrates the utility of such an approach, as we were not only able to screen a large number of conditions of small molecule activators and inhibitors of Wnt signaling, but also observe the impacts of paracrine signalling during osteogenesis, an outcome that would be otherwise invisible under standard culture conditions. Furthermore, we were able to demonstrate that information gained from the MBA was not only valid when transferred back to static conditions, but could inform further experiments. Significantly, this finding also indicates that this MBA screening strategy has significant potential to be used to efficiently generate data useful in improving MPC osteogenic differentiation.

More specifically, whilst we confirmed the requirement for both canonical and non-canonical Wnt signalling during osteogenesis (through our use of IWR-1 and IWP-4 Wnt inhibitors), we show the somewhat confounding effects of CHIR (a small molecule Wnt agonist) upon osteogenesis and gain some insights into the manner by which it strongly inhibits differentiation, when in the presence of dexamethasone. We suggest that, although CHIR acts, as expected, to activate Wnt signalling and subsequently increase expression of key osteogenic transcription factors (*RUNX2*, *MSX2* and *DLX5*), the decrease in *ALP* and *SPARC* expression leads to an overall block of differentiation.

The strategy utilised in this study can be similarly applied in the elucidation of distinct factor treatments, other differentiation lineages, or even other cell types, to provide useful data with which to both gain new fundamental insights and to optimise culture conditions in developing methods of cellular differentiation for therapeutic applications.

## Supporting Information

Figure S1 **Characterisation of MPC donors.** A Graph summarizing results of flow cytometric analysis of surface antigen expression in MPCs from donor 1 and 2. B Tri-lineage differentiation of MPCs from donors 1 and 2. Images show Alizarin red, Oil red O and Alcian blue staining of osteogenic, adipogenic and chondrogenic cultures respectively. Cultures were analysed after 21 days in differentiation medium with growth medium as a control. Scale = 100 µm.
(TIF)

Figure S2 **Microbioreactor array design and validation.** A Microbioreactor array design and key features. B Schematic of device assembly. *Via* holes join microchannel structures between PDMS layers 1 and 2. C Design normalised concentrations of factors in each column, corresponding to panels E and F. Stock factor and buffer solutions are provided at normalised concentrations of 3 and 0, respectively, to allow for subsequent dilution. D Photograph of microbioreactor array filled with red, yellow and blue food dyes (representing factors A1, B1 & C1, respectively), and mixed with PBS (buffers A0, B0, & C0). E Fluorimetric quantification of soluble factor levels in each column. Stock solution of 40 kDa FITC-dextran was provided at 100 µM, therefore the design concentration levels are 0, 16.7 and 33.3 µM. Bars represent mean ± SD of 2 independently fabricated devices. Modified from D. M. Titmarsh, J. E. Hudson, A. Hidalgo, A. G. Elefanty, E. G. Stanley, E. J. Wolvetang, J. J. Cooper-White, Microbioreactor Arrays for Full Factorial Screening of Exogenous and Paracrine Factors in Human Embryonic Stem Cell Differentiation. *PLoS ONE* 2012, *7*. e52405, DOI: 10.1371/journal.pone.0052405.
(TIF)

Figure S3 **Microbioreactor array screening of Wnt modulation in MPC osteogenesis - Donor 1 Run 1.** A Panel of screening conditions in microbioreactor arrays. B Confocal microscopy images of endpoint PI (DNA) and ELF97 (alkaline phosphatase activity) staining from a representative experiment. Direction of fluid flow was from top to bottom. C Heatmaps of expression indices for DNA, ELF97, and ELF97/DNA ratio. D Main effects plot showing effect of DONOR, CHIR99021 (CHIR), IWP-4, IWR-1 and POSITION on expression indices for DNA, ELF97, and ELF97/DNA ratio. E Interaction effects plot showing effects of 2 combined factors on DNA, ELF97, and ELF97/DNA ratio.
(TIF)

Figure S4 **Microbioreactor array screening of Wnt modulation in MPC osteogenesis - Donor 1 Run 2.** A Panel of screening conditions in microbioreactor arrays. B Confocal microscopy images of endpoint PI (DNA) and ELF97 (alkaline phosphatase activity) staining from a representative experiment. Direction of fluid flow was from top to bottom. C Heatmaps of expression indices for DNA, ELF97, and ELF97/DNA ratio. D Main effects plot showing effect of DONOR, CHIR99021 (CHIR), IWP-4, IWR-1 and POSITION on expression indices for DNA, ELF97, and ELF97/DNA ratio. E Interaction effects plot showing effects of 2 combined factors on DNA, ELF97, and ELF97/DNA ratio.
(TIF)

Figure S5 **Microbioreactor array screening of Wnt modulation in MPC osteogenesis - Donor 2 Run 1.** A Panel of screening conditions in microbioreactor arrays. B Confocal microscopy images of endpoint PI (DNA) and ELF97 (alkaline phosphatase activity) staining from a representative experiment. Direction of fluid flow was from top to bottom. C Heatmaps of expression indices for DNA, ELF97, and ELF97/DNA ratio. D Main effects plot showing effect of DONOR, CHIR99021 (CHIR), IWP-4, IWR-1 and POSITION on expression indices for DNA, ELF97, and ELF97/DNA ratio. E Interaction effects plot showing effects of 2 combined factors on DNA, ELF97, and ELF97/DNA ratio.
(TIF)

Figure S6 **Microbioreactor array screening of Wnt modulation in MPC osteogenesis - Donor 2 Run 2.** A Panel of screening conditions in microbioreactor arrays. B Confocal microscopy images of endpoint PI (DNA) and ELF97 (alkaline phosphatase activity) staining from a representative experiment. Direction of fluid flow was from top to bottom. C Heatmaps of expression indices for DNA, ELF97, and ELF97/DNA ratio. D Main effects plot showing effect of DONOR, CHIR99021 (CHIR), IWP-4, IWR-1 and POSITION on expression indices for DNA, ELF97, and ELF97/DNA ratio. E Interaction effects plot showing effects of 2 combined factors on DNA, ELF97, and ELF97/DNA ratio.
(TIF)

Figure S7 **Comparative expression indices between runs.** Heatmaps of expression indices for DNA, ELF97, and ELF97/DNA ratio. 270 individual chambers from bioreactors are paired with corresponding chambers for 2 runs from each of 2 MPC donors. The average of all 4 runs is also shown.
(TIF)

Figure S8 **Factorial analysis of pooled data.** A Main effects plots rating effect magnitudes of DONOR, CHIR99021 (µM), IWP-4 (µM), IWR-1 (µM) and POSITION (Row) on expression indices for DNA, ELF97 and ELF97/DNA. B Interaction effects plots showing effect magnitudes of combinations of two stimuli on expression indices for DNA, ELF97 and ELF97/DNA. In all graphs the average response of all 4 runs is shown.
(TIF)

Figure S9 **Dose response of CHIR.** MPCs were treated with various concentrations of CHIR for 7 days and EC50 was determined by performing alkaline phosphatase activity assay (EC_50_ = 0.631 µM).
(TIF)

Figure S10 **Fast Blue Staining of Cells Grown In Microbioreactor Array.** Confirmation of alkaline phosphatase activity and row-dependency with Fast Blue stain. Diameter of chambers shown is ∼1.63 mm.
(TIF)

Table S1 **Microbioreactor Array Physical parameters.**
(DOCX)
