# Supplementary figures and images for: Microbioreactor Array Screening of Wnt Modulators and Microenvironmental Factors in Osteogenic Differentiation of Mesenchymal Progenitor Cells
Source: PLoS One. 2013 Dec 23;8(12):e82931. doi: 10.1371/journal.pone.0082931 (PMC3871528; doi:10.1371/journal.pone.0082931)

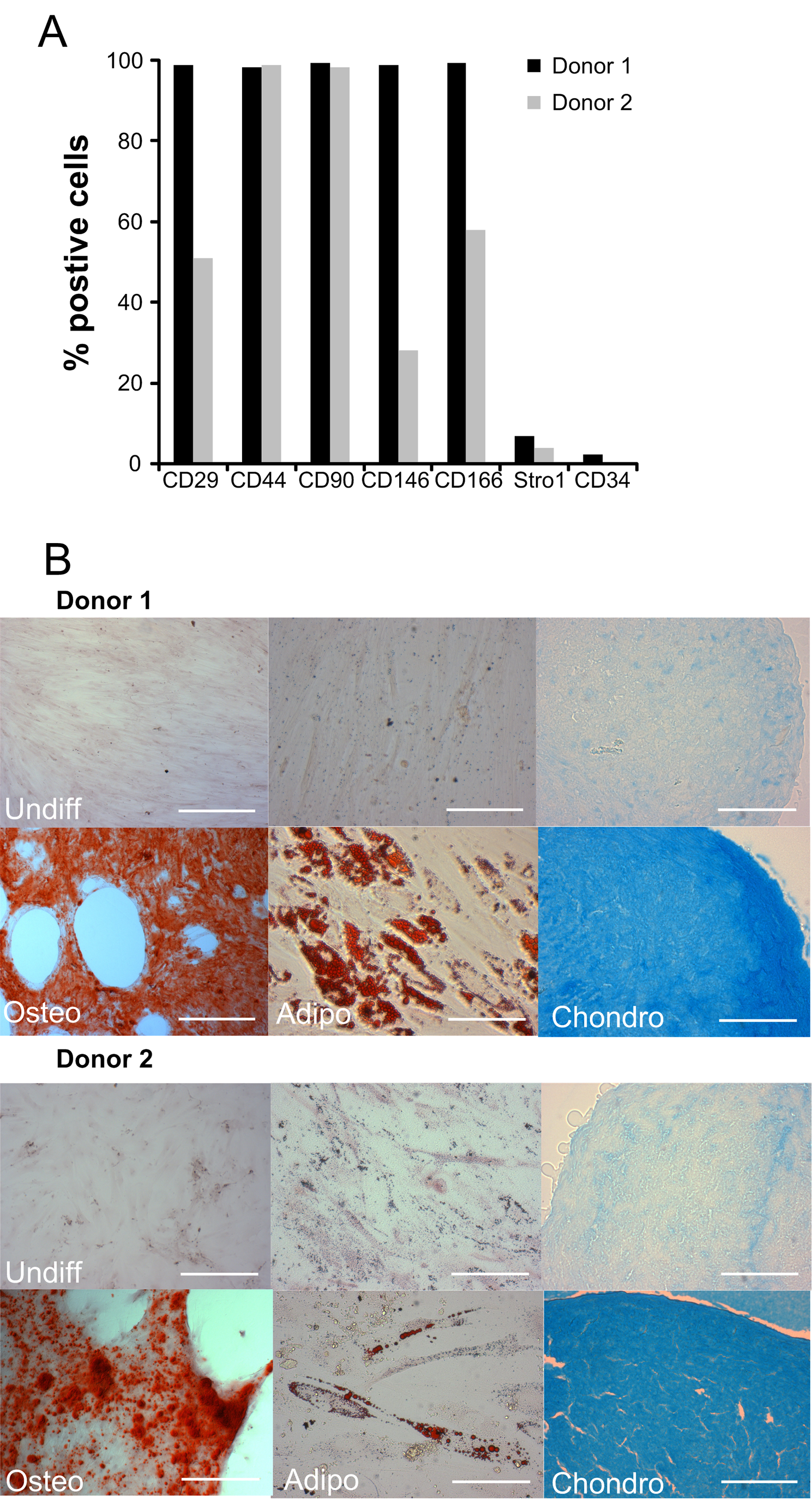

Supplement: Figure S1 — Characterisation of MPC donors. A Graph summarizing results of flow cytometric analysis of surface antigen expression in MPCs from donor 1 and 2. B Tri-lineage differentiation of MPCs from donors 1 and 2. Images show Alizarin red, Oil red O and Alcian blue staining of osteogenic, adipogenic and chondrogenic cultures respectively. Cultures were analysed after 21 days in differentiation medium with growth medium as a control. Scale = 100 µm. (TIF) [file pone.0082931.s001.tif]

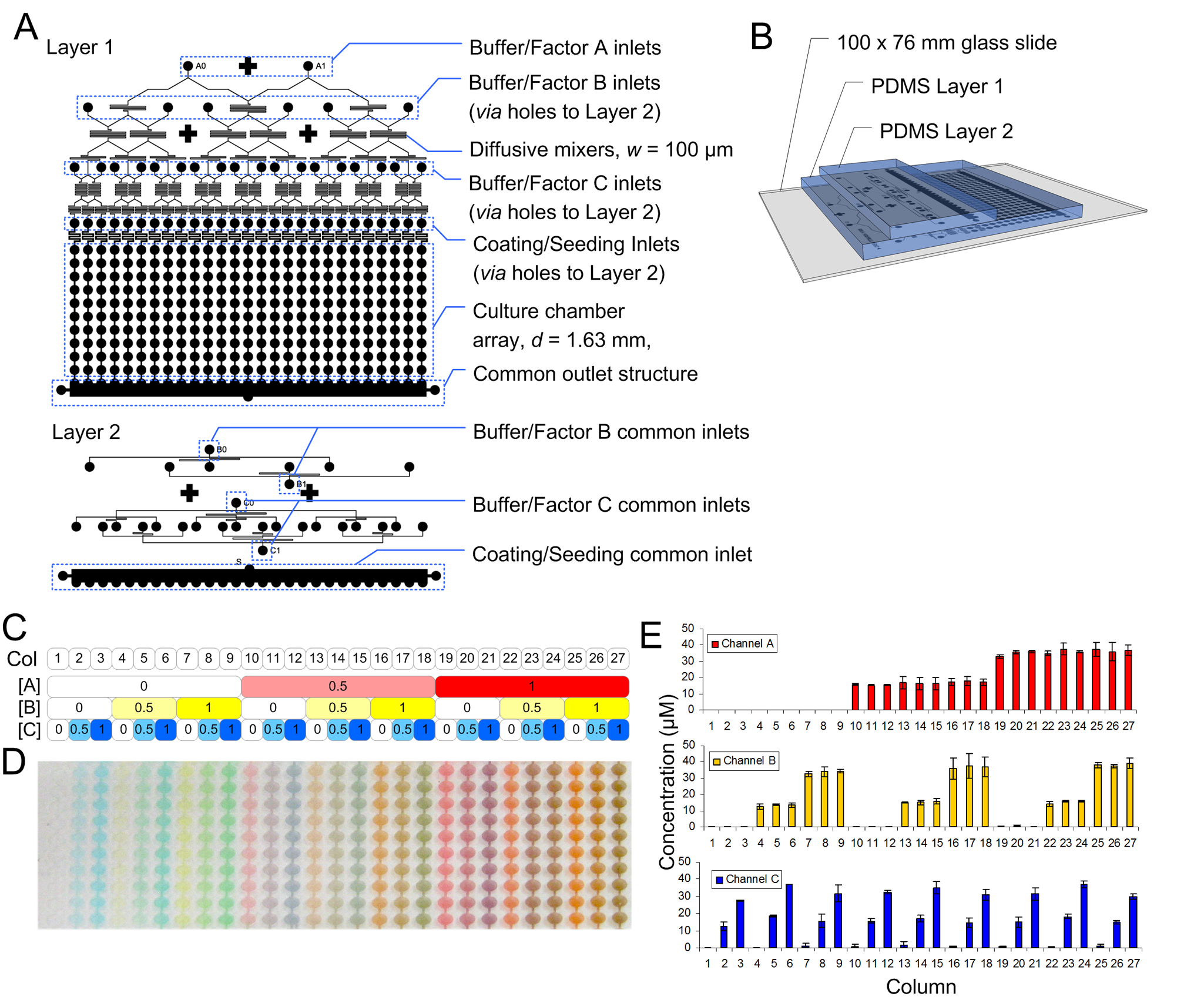

Supplement: Figure S2 — Microbioreactor array design and validation. A Microbioreactor array design and key features. B Schematic of device assembly. Via holes join microchannel structures between PDMS layers 1 and 2. C Design normalised concentrations of factors in each column, corresponding to panels E and F. Stock factor and buffer solutions are provided at normalised concentrations of 3 and 0, respectively, to allow for subsequent dilution. D Photograph of microbioreactor array filled with red, yellow and blue food dyes (representing factors A1, B1 & C1, respectively), and mixed with PBS (buffers A0, B0, & C0). E Fluorimetric quantification of soluble factor levels in each column. Stock solution of 40 kDa FITC-dextran was provided at 100 µM, therefore the design concentration levels are 0, 16.7 and 33.3 µM. Bars represent mean ± SD of 2 independently fabricated devices. Modified from D. M. Titmarsh, J. E. Hudson, A. Hidalgo, A. G. Elefanty, E. G. Stanley, E. J. Wolvetang, J. J. Cooper-White, Microbioreactor Arrays for Full Factorial Screening of Exogenous and Paracrine Factors in Human Embryonic Stem Cell Differentiation. PLoS ONE 2012, 7. e52405, DOI: 10.1371/journal.pone.0052405. (TIF) [file pone.0082931.s002.tif]

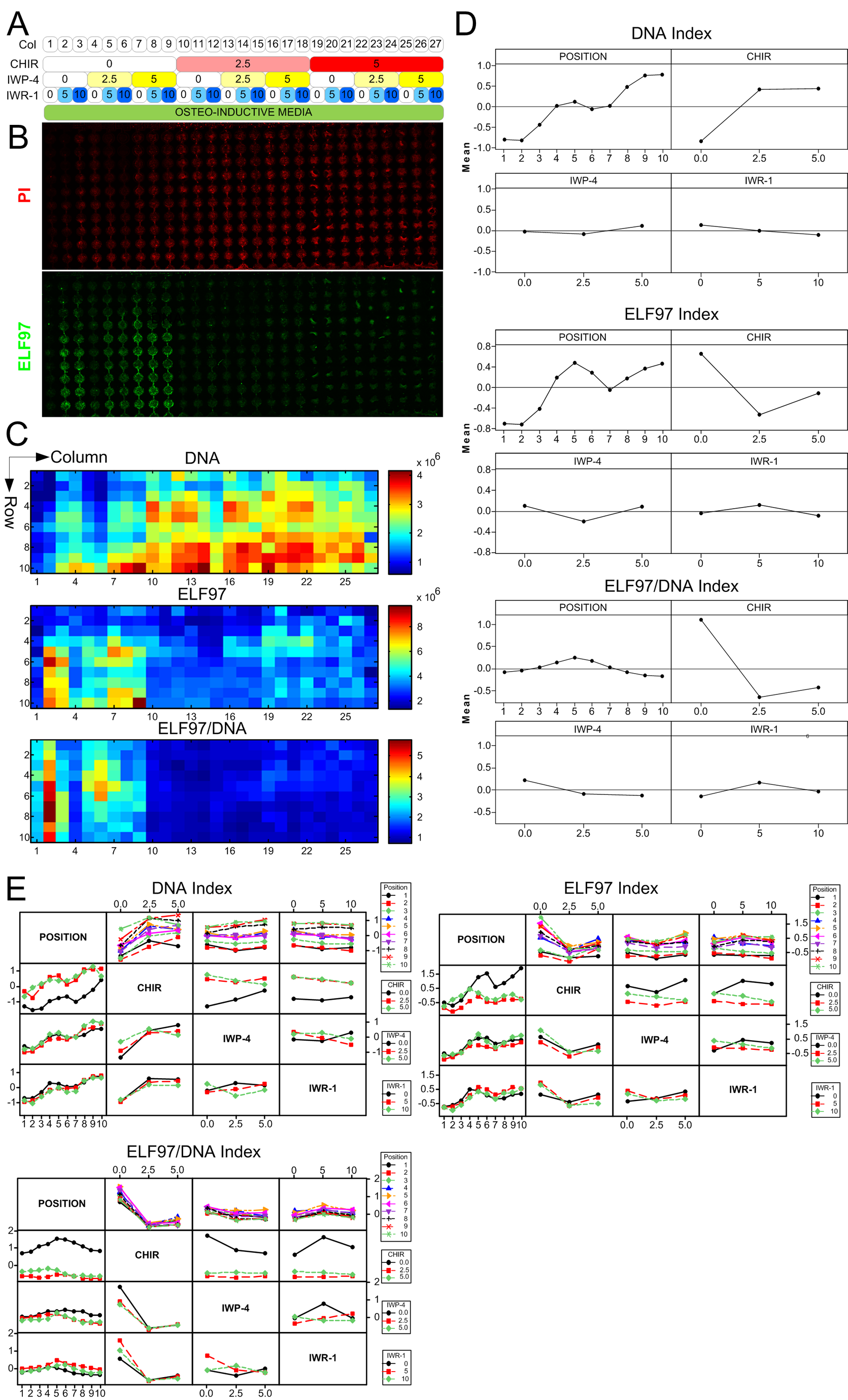

Supplement: Figure S3 — Microbioreactor array screening of Wnt modulation in MPC osteogenesis - Donor 1 Run 1. A Panel of screening conditions in microbioreactor arrays. B Confocal microscopy images of endpoint PI (DNA) and ELF97 (alkaline phosphatase activity) staining from a representative experiment. Direction of fluid flow was from top to bottom. C Heatmaps of expression indices for DNA, ELF97, and ELF97/DNA ratio. D Main effects plot showing effect of DONOR, CHIR99021 (CHIR), IWP-4, IWR-1 and POSITION on expression indices for DNA, ELF97, and ELF97/DNA ratio. E Interaction effects plot showing effects of 2 combined factors on DNA, ELF97, and ELF97/DNA ratio. (TIF) [file pone.0082931.s003.tif]

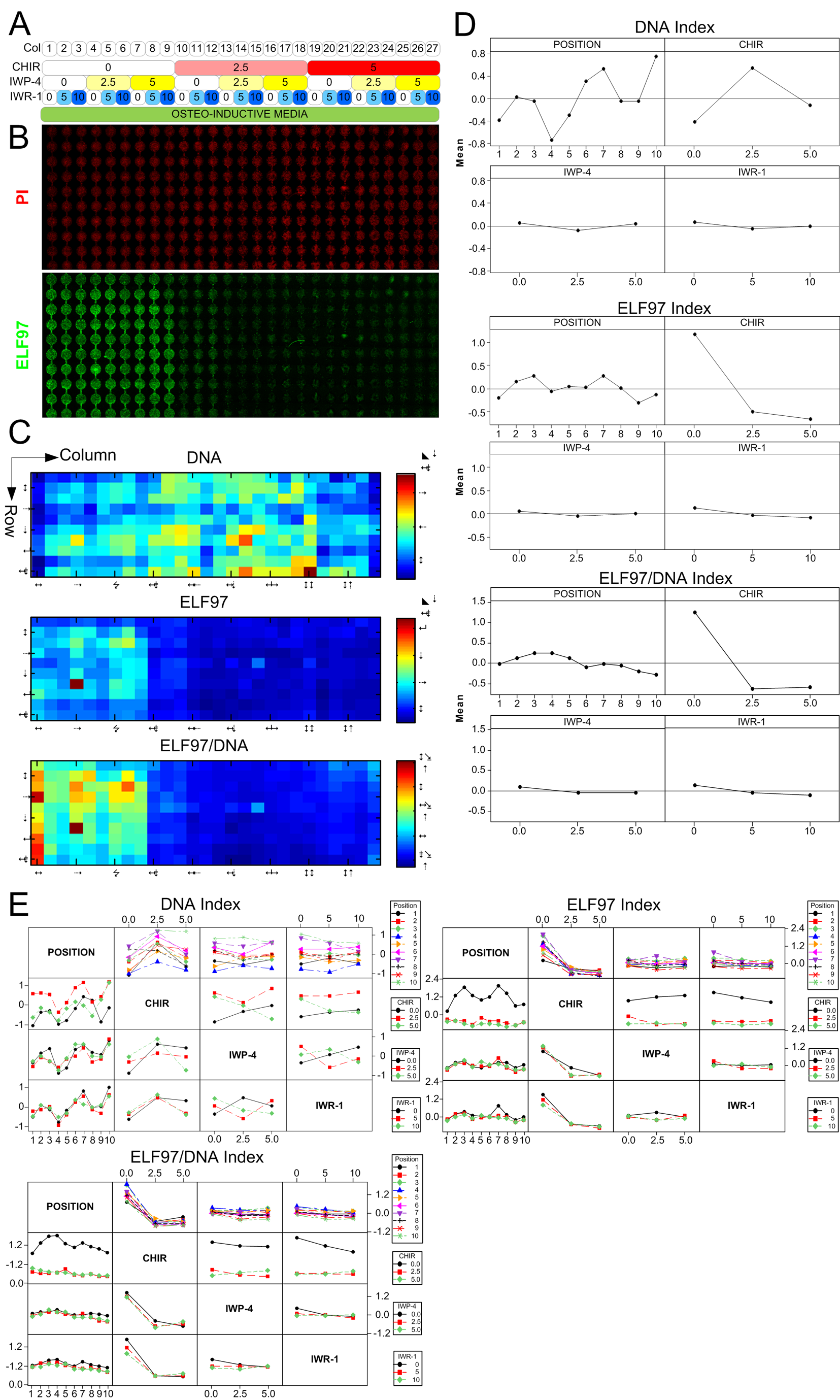

Supplement: Figure S4 — Microbioreactor array screening of Wnt modulation in MPC osteogenesis - Donor 1 Run 2. A Panel of screening conditions in microbioreactor arrays. B Confocal microscopy images of endpoint PI (DNA) and ELF97 (alkaline phosphatase activity) staining from a representative experiment. Direction of fluid flow was from top to bottom. C Heatmaps of expression indices for DNA, ELF97, and ELF97/DNA ratio. D Main effects plot showing effect of DONOR, CHIR99021 (CHIR), IWP-4, IWR-1 and POSITION on expression indices for DNA, ELF97, and ELF97/DNA ratio. E Interaction effects plot showing effects of 2 combined factors on DNA, ELF97, and ELF97/DNA ratio. (TIF) [file pone.0082931.s004.tif]

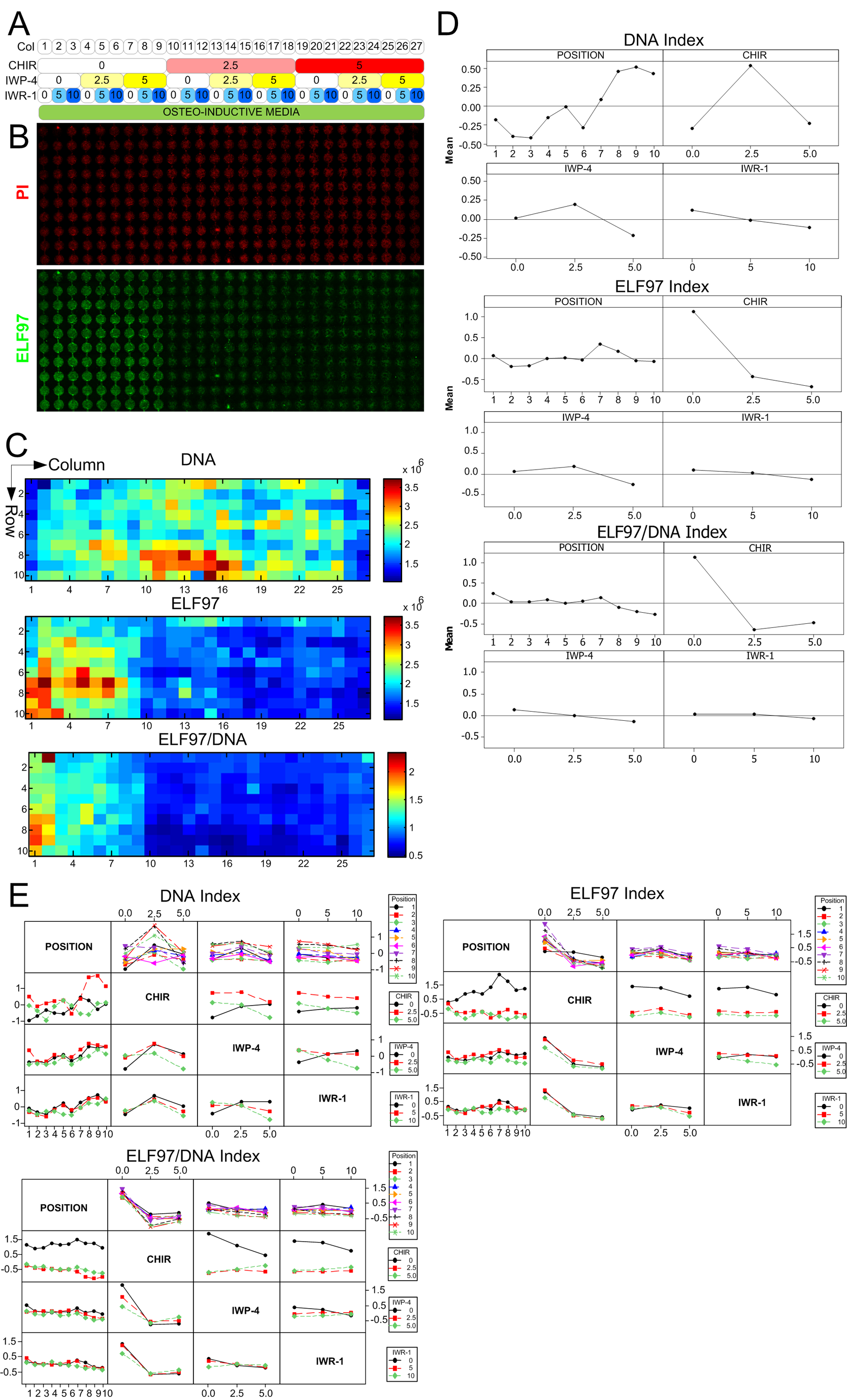

Supplement: Figure S5 — Microbioreactor array screening of Wnt modulation in MPC osteogenesis - Donor 2 Run 1. A Panel of screening conditions in microbioreactor arrays. B Confocal microscopy images of endpoint PI (DNA) and ELF97 (alkaline phosphatase activity) staining from a representative experiment. Direction of fluid flow was from top to bottom. C Heatmaps of expression indices for DNA, ELF97, and ELF97/DNA ratio. D Main effects plot showing effect of DONOR, CHIR99021 (CHIR), IWP-4, IWR-1 and POSITION on expression indices for DNA, ELF97, and ELF97/DNA ratio. E Interaction effects plot showing effects of 2 combined factors on DNA, ELF97, and ELF97/DNA ratio. (TIF) [file pone.0082931.s005.tif]

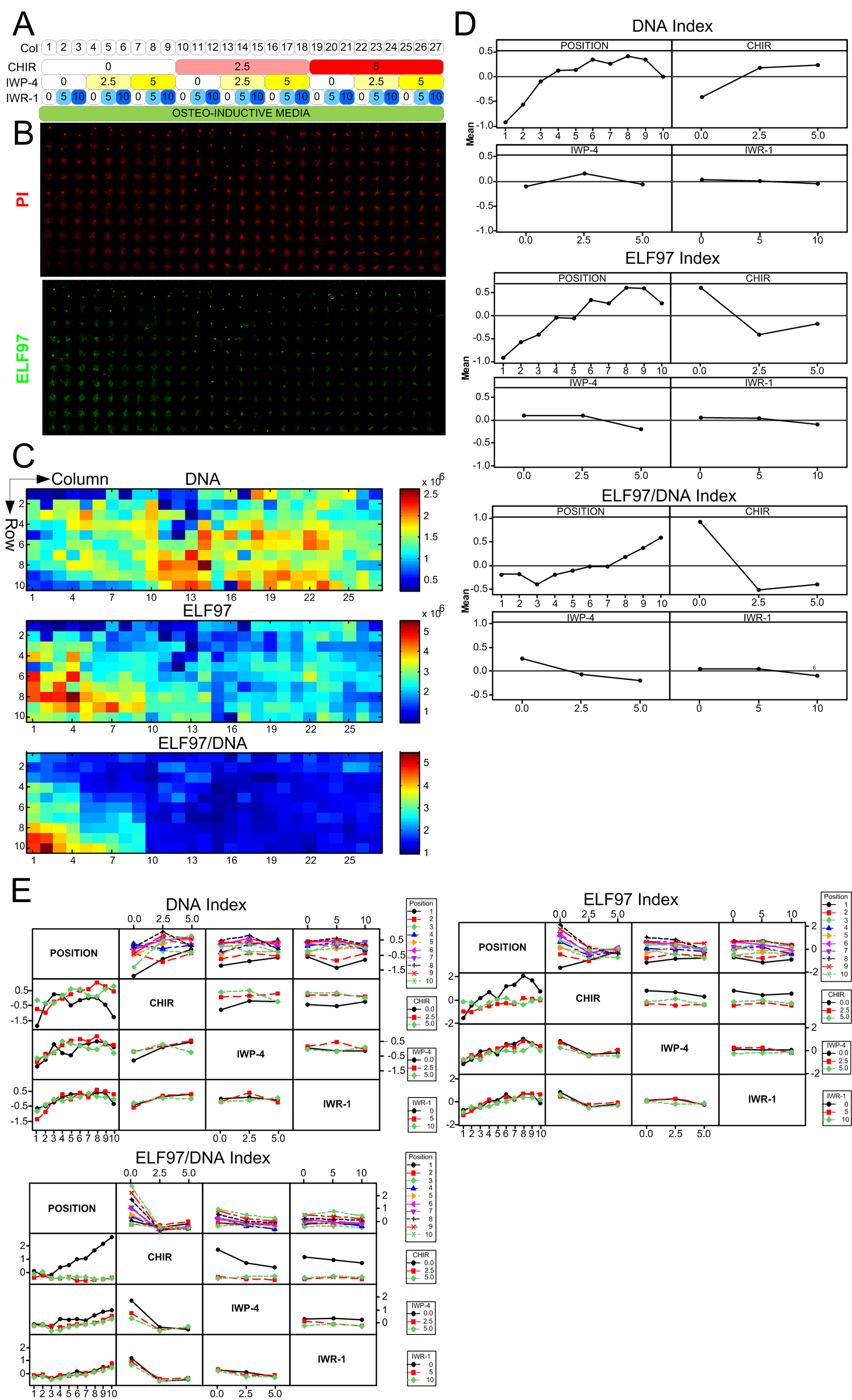

Supplement: Figure S6 — Microbioreactor array screening of Wnt modulation in MPC osteogenesis - Donor 2 Run 2. A Panel of screening conditions in microbioreactor arrays. B Confocal microscopy images of endpoint PI (DNA) and ELF97 (alkaline phosphatase activity) staining from a representative experiment. Direction of fluid flow was from top to bottom. C Heatmaps of expression indices for DNA, ELF97, and ELF97/DNA ratio. D Main effects plot showing effect of DONOR, CHIR99021 (CHIR), IWP-4, IWR-1 and POSITION on expression indices for DNA, ELF97, and ELF97/DNA ratio. E Interaction effects plot showing effects of 2 combined factors on DNA, ELF97, and ELF97/DNA ratio. (TIF) [file pone.0082931.s006.tif]

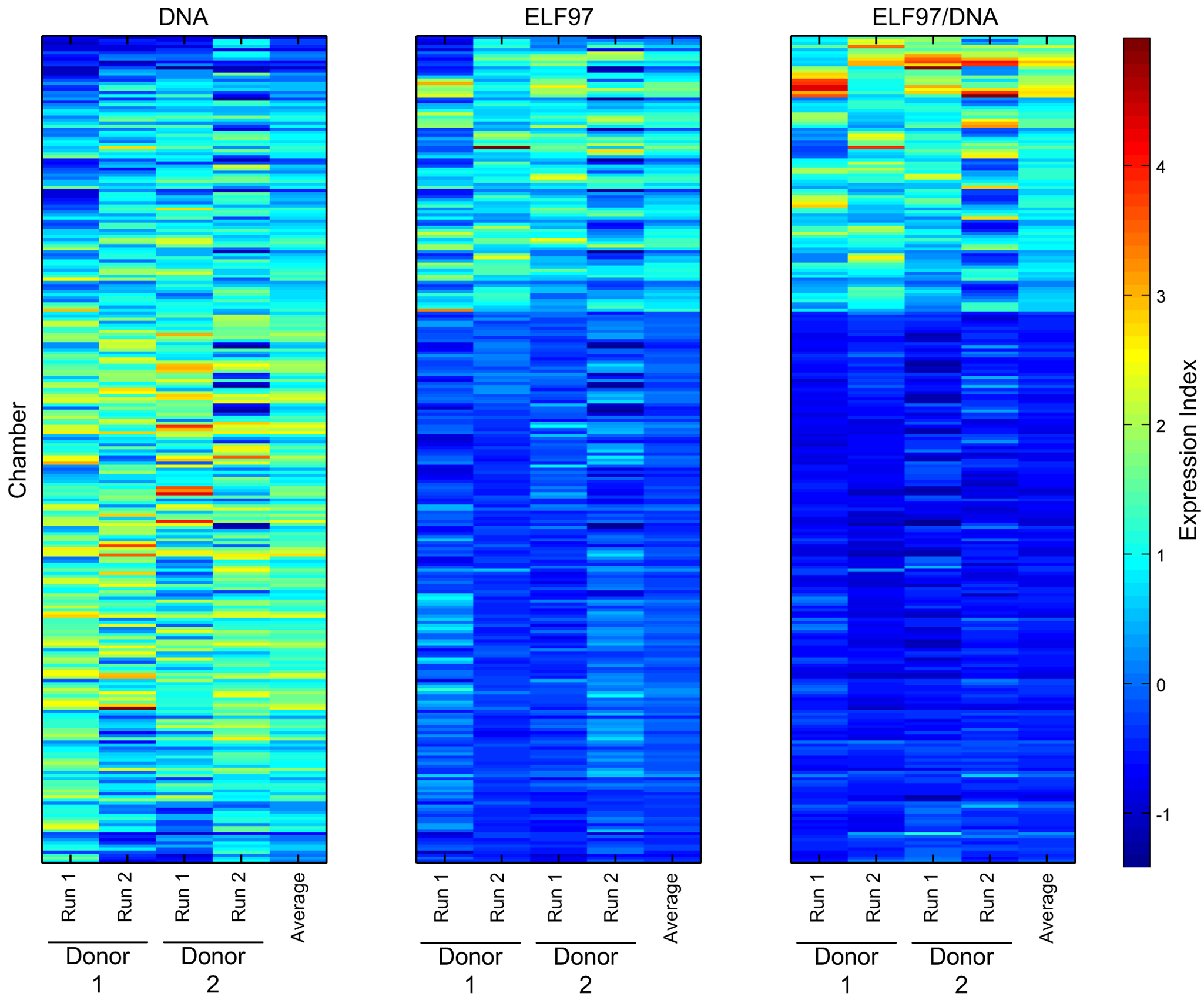

Supplement: Figure S7 — Comparative expression indices between runs. Heatmaps of expression indices for DNA, ELF97, and ELF97/DNA ratio. 270 individual chambers from bioreactors are paired with corresponding chambers for 2 runs from each of 2 MPC donors. The average of all 4 runs is also shown. (TIF) [file pone.0082931.s007.tif]

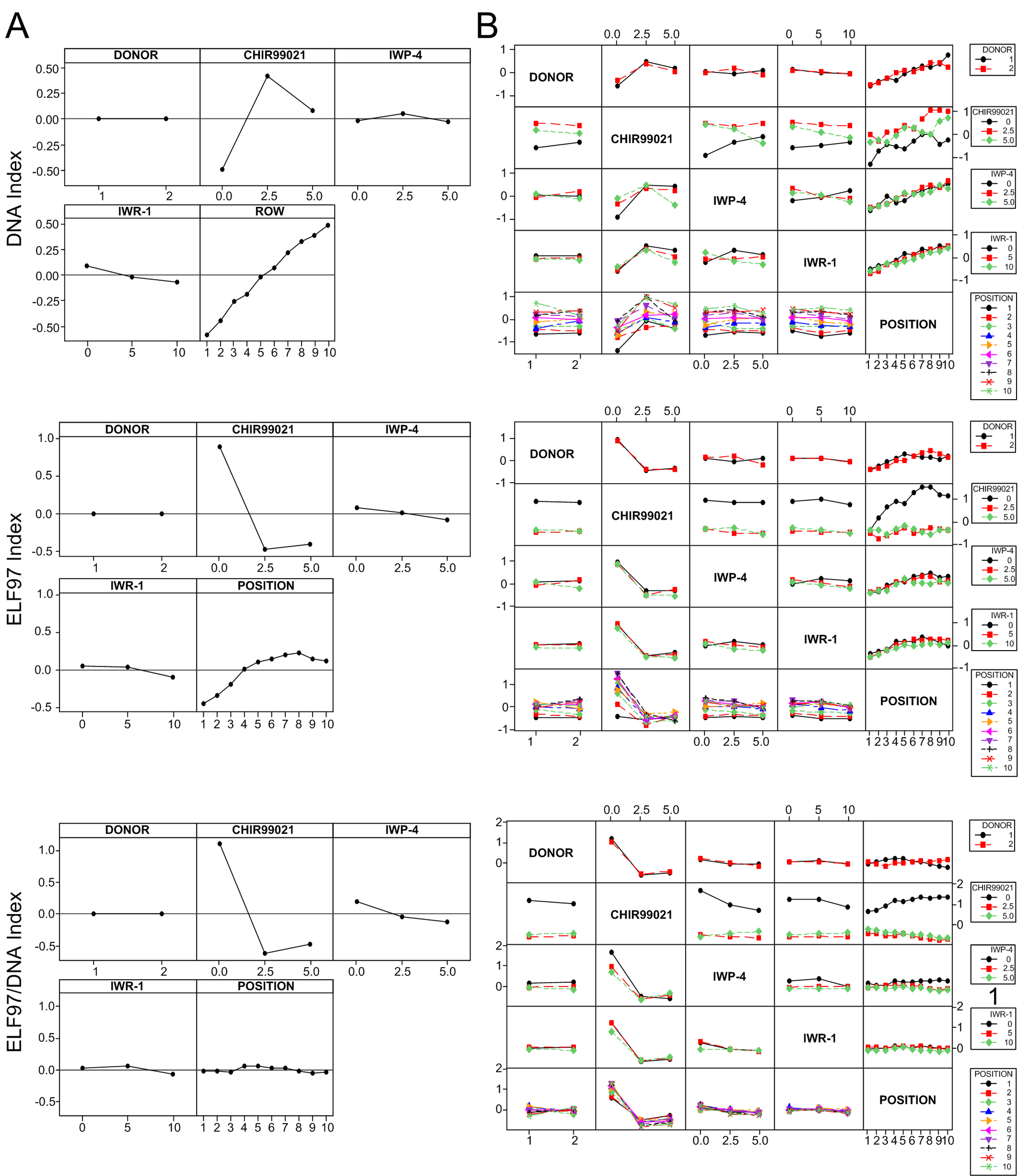

Supplement: Figure S8 — Factorial analysis of pooled data. A Main effects plots rating effect magnitudes of DONOR, CHIR99021 (µM), IWP-4 (µM), IWR-1 (µM) and POSITION (Row) on expression indices for DNA, ELF97 and ELF97/DNA. B Interaction effects plots showing effect magnitudes of combinations of two stimuli on expression indices for DNA, ELF97 and ELF97/DNA. In all graphs the average response of all 4 runs is shown. (TIF) [file pone.0082931.s008.tif]

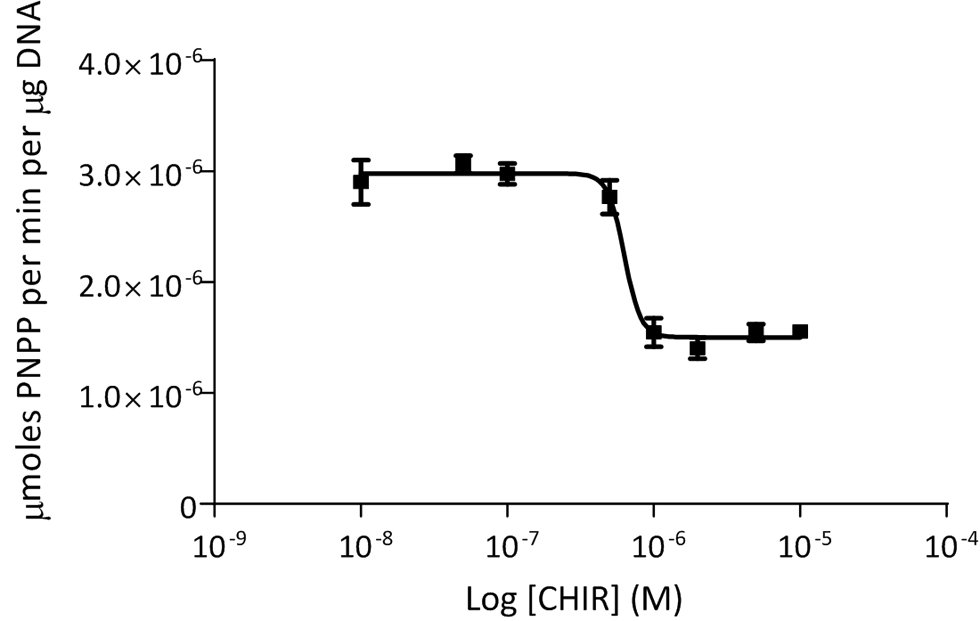

Supplement: Figure S9 — Dose response of CHIR. MPCs were treated with various concentrations of CHIR for 7 days and EC50 was determined by performing alkaline phosphatase activity assay (EC50 = 0.631 µM). (TIF) [file pone.0082931.s009.tif]

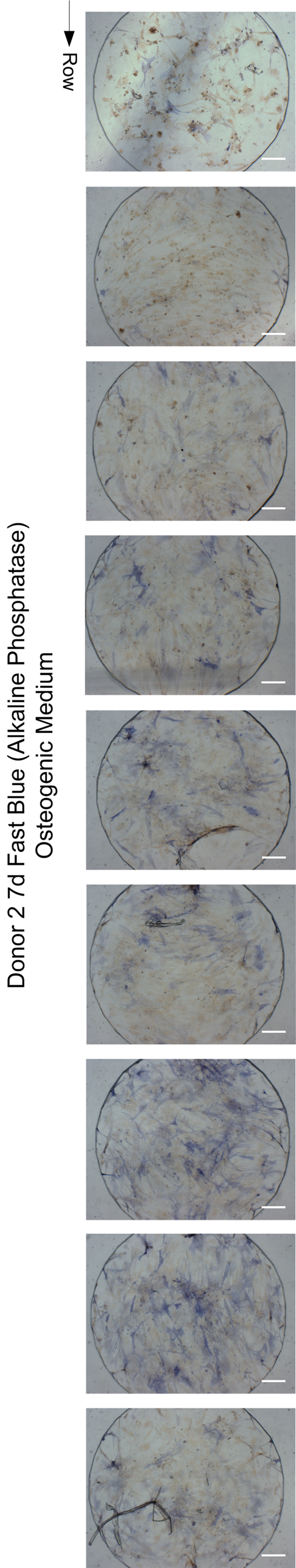

Supplement: Figure S10 — Fast Blue Staining of Cells Grown In Microbioreactor Array. Confirmation of alkaline phosphatase activity and row-dependency with Fast Blue stain. Diameter of chambers shown is ∼1.63 mm. (TIF) [file pone.0082931.s010.tif]
