# Supplementary material for: Microbioreactor Array Screening of Wnt Modulators and Microenvironmental Factors in Osteogenic Differentiation of Mesenchymal Progenitor Cells
Source: PLoS One. 2013 Dec 23;8(12):e82931. doi: 10.1371/journal.pone.0082931 (PMC3871528; doi:10.1371/journal.pone.0082931)
Supplement: Table S1 — Microbioreactor Array Physical parameters. (DOCX) [file pone.0082931.s011.docx]

Physical Parameters

| **Parameter** | **Unit** | **Microbioreactor** | **24-well Plate** |
| --- | --- | --- | --- |
| **Chamber Dimensions** |  |  |  |
| Diameter  Culture Area Culture Area Specified Height  Nominal Volume  Surface-Area-to-Volume (SAV) Ratio  Plate to Bioreactor Concentration Factor | mm mm2 mm2 mm μL  mm2/mm3  - | 1.63  2.09  -  0.1  0.21  12  25 | 15.49  188.4  200  2.5  500  0.4  1 |
| **Column Dimensions** |  |  |  |
| Culture Area  Volume | mm2  μL | 21.4  2.14 |  |
| **Array Dimensions** |  |  |  |
| Culture Area  Volume | mm2  μL | 579  58 |  |
| **Nominal Flow Conditions** |  |  |  |
| Culture Area per Column Flowrate per Unit Area Channel Flowrate  Total Array Flowrate | cm2 μL/h/cm2 μL/h  μL/h | 0.214  6.22  1.33  36 |  |
| **a Reynolds Number, Re** |  |  |  |
| Fluid Density, ρ  Average Velocity, *Q*/*A* (chamber at full width) Average Velocity, *Q*/*A* (interconnect) Hydraulic Diameter, *Dh* (full width) Hydraulic Diameter, *Dh* (interconnect) Fluid Viscosity, µ  Average Re (chamber at full width)  Average Re (interconnect) | kg/m3 m/s m/s m  m  Pa.s  -  - | 1000  9.08E-07  5.92E-06  1.88E-04  1.43E-04  1.00E-03  1.71E-04  8.47E-04 |  |
| **b Péclet Number, Pe** |  |  |  |
| Diffusivity (glucose) Diffusivity (40kDa growth factor)  Average Pe (chamber at full width) (glucose)  Average Pe (chamber at full width) (GF) | m2/s m2/s  -  - | 6.00E-10  8.00E-11  0.285  2.14 |  |
| **c Shear Stress, τ** |  |  |  |
| Shear stress (chamber at full width)  Shear stress (interconnect) | Pa  Pa | 1.36E-04  8.89E-04 |  |

**Notes**

a Calculated as Re = ρ*QD*

*h*

µ*A* , where density (ρ) = 1×103 kg/m3; *Q* represents flowrate (m3/s); *D*

*h*

represents hydraulic

diameter; viscosity (µ) = 10-3 Pa.s; and *A* represents cross-sectional area. Density and viscosity assumed as for water.

b Calculated as Pe = *QD*

*h*

*DAB*

*A* , where *DAB*

represents the diffusion coefficient.

c Shear stress estimated as τ = 6µ*Q*

*h*2 *w* , where height (*h*) *=* 1×10-4 m; and width (*w*) *=* 1.63×10-3 m for chamber at full

width and 2.5×10-4 m for chamber interconnect.
